# Feasibility, acceptability, and preliminary efficacy protocol of an intervention for caregivers of hospice patients living with dementia: A pilot randomized control trial

**DOI:** 10.1371/journal.pone.0332360

**Published:** 2025-11-03

**Authors:** Ebtesam Tamer, Donald R. Hoover, Margaret V. McDonald, Kira G. Sheldon, Felix Vasquez, Maryam Gaibi, Elizabeth A. Luth

**Affiliations:** 1 School of Public Health, Rutgers University, Piscataway, New Jersey, United States of America; 2 Institute for Health, Health Care Policy and Aging Research, Rutgers University, New Brunswick, New Jersey, United States of America; 3 Department of Statistics, Rutgers University, New Brunswick, New Jersey, United States of America; 4 Center for Home Care Policy & Research, VNS Health, New York, New York, United States of America; 5 VNS Health Hospice Care, New York, New York, United States of America; 6 Department of Family Medicine and Community Health, Rutgers University, New Brunswick, New Jersey, United States of America; Uttara Adhunik Medical College, BANGLADESH

## Abstract

**Introduction:**

Caring for dying persons living with dementia (PLwD) poses substantial challenges for family care partners (FCPs), who often experience significant emotional and physical strain. Home hospice provides support to enable home death, including support for FCPs. We are pilot testing a home hospice intervention (Enhancing Dementia Instruction and Tool in Home Hospice Care (EDITH-HC)) to reduce FCP burden and improve care for PLwD. This pilot test assesses feasibility, acceptability, and preliminary efficacy using a non-blinded randomized clinical trial (RCT).

**Methods:**

We aim to match 24 hospice nurses and social workers (“clinicians”) with 96 FCPs of home hospice PLwD. Clinician teams are randomized to the intervention or control condition using an adaptive randomized strategy to maintain balance between Black and White FCPs in each study arm. The intervention consists of educational videos to enhance clinicians’ knowledge about and confidence in providing dementia-specific end-of-life care and a tool for clinicians to use with FCPs to identify and address FCPs’ dementia-related stressors. Participants answer online or telephone surveys at baseline, following each of four routine home visits, and post-intervention. We will compile descriptive statistics to report on feasibility and acceptability measures. We will conduct t-tests of differences and linear regression analyses to examine differences in FCP burden (primary outcome) between baseline and first subsequent clinician visit (primary time point). Additional exploratory analyses are explained.

**Results:**

We have enrolled 29 clinicians and 53 FCPs and aim to have 24 clinicians matched with 96 FCPs (N = 120) to complete the intervention.

**Discussion:**

If the pilot shows positive results for the intervention, we plan to test it in a multi-site, fully-powered RCT. Enhancing support for clinicians and FCP of PLwD could optimize FCP and hospice patient outcomes.

## Introduction

Caring for persons living with dementia (PLwD) nearing the end of life can be an overwhelming experience for family care partners (FCPs) [1,2]. FCPs are family members, friends, or other significant individuals who provide unpaid care, often navigating the complexities of managing daily activities, medical needs, and behavioral symptoms of persons with serious illnesses, including dementia. Dementia care is complex and emotionally taxing, often leading to significant stress and burden for FCPs [3]. Studies have shown that FCPs of PLwD experience high levels of emotional distress and burnout, which can adversely affect their health and the quality of care provided to the PLwD [4,5]. This can be particularly burdensome for Black caregivers, as prior research highlights that the intersection of caregiving stress and racial discrimination can exacerbate mental health issues, [6] including depression and anxiety [7]. This can affect both the caregiver’s well-being and their ability to provide care effectively. Despite the critical role FCPs play in end-of-life care, there is a lack of targeted support, [8,9] and educational resources designed to address their specific challenges in the home hospice setting [10]. Qualitative research highlights the critical role of effective communication and education provided by clinicians to family caregivers [11].

Home hospice care provides physical, psychological, and emotional support to individuals with a life expectancy of 6 months or less—as certified by two physicians—and their families [12]. Home hospice is provided in private residences, nursing homes, and assisted living facilities [12]. Because home hospice nurses and social workers have ongoing relationships with hospice patients and their families, home hospice is an appropriate environment for additional support targeting the needs of PLwD and FCPs [13]. One exception is the Aliviado Dementia Care intervention, which targets advanced dementia symptom management in the hospice population [14]. To our knowledge, none currently provide direct support to their FCPs, [15] highlighting a significant area for improvement.

The Enhanced Dementia Instruction and Tool in Home Hospice Care (EDITH-HC) project aims to fill this gap by developing and pilot testing an intervention specifically designed to support Black and White FCPs of PLwD in home hospice. The EDITH-HC intervention consists of two components: i) educational videos tailored for hospice clinicians to enhance their understanding and management of dementia-specific issues, and ii) an assessment tool to identify and address the unique stressors FCPs face [16]. EDITH-HC aims to improve the overall quality of dementia care in home hospice settings by focusing on how clinicians can tailor support for FCPs with the idea that with enhanced support, care for PLwD will get better [17].

This study is a feasibility, acceptability, and pilot randomized clinical trial (RCT) of the EDITH-HC intervention versus usual care in a sample of 24 clinicians and 96 FCPs (12 clinicians and 48 FCP in the intervention and control groups). The sample size was selected to ensure sufficient variability in engagement, adherence, and feedback to assess feasibility, fidelity, and acceptability [18]. These numbers are based on the size of the clinician recruitment pool at the participating hospice agency. A minimum of 10 participants in each group is recommended for pilot acceptability and feasibility studies [19]. FCP recruitment reflects an intention to match each enrolled clinician with four FCP on their caseload to obtain variability in feedback. The sample size will provide the amount of data necessary to inform recruitment processes, evaluate intervention delivery, and guide sample size calculation for future, fully-powered trials. Our study aims to assess: 1) the feasibility and acceptability for clinicians and FCPs of introducing the EDITH-HC intervention into existing clinical workflows in-home hospice care; 2) the preliminary efficacy of the intervention in reducing FCP caregiving burden; 3) whether the intervention increases FCP self-efficacy and caregiving preparedness, increases clinician dementia knowledge, and reduces live discharge among hospice patients [16]. Live discharge occurs when a patient leaves hospice before dying and happens 15%−17% of the time [20] and more frequently among PLwD [21,22]. Live discharge is considered a disruptive and burdensome outcome for patients and families in general [23,24] and for PLwD in particular [25,26]. From this study, we expect that preliminary findings will indicate that the intervention is feasible and well-received by participants. We will also investigate whether the intervention significantly reduces FCP burden, increases FCP self-efficacy and preparedness, enhances clinician knowledge regarding dementia caregiving, and decreases live discharges among hospice patients whose FCPs are enrolled in the study.

This paper describes the protocol and status of our ongoing pilot study of the EDITH-HC intervention. Through this study, we aim to provide evidence for an intervention that can enhance the quality of life for both PLwD and their FCPs, improving dementia care in home hospice settings.

## Methods

### Study design

**Fig 1:**
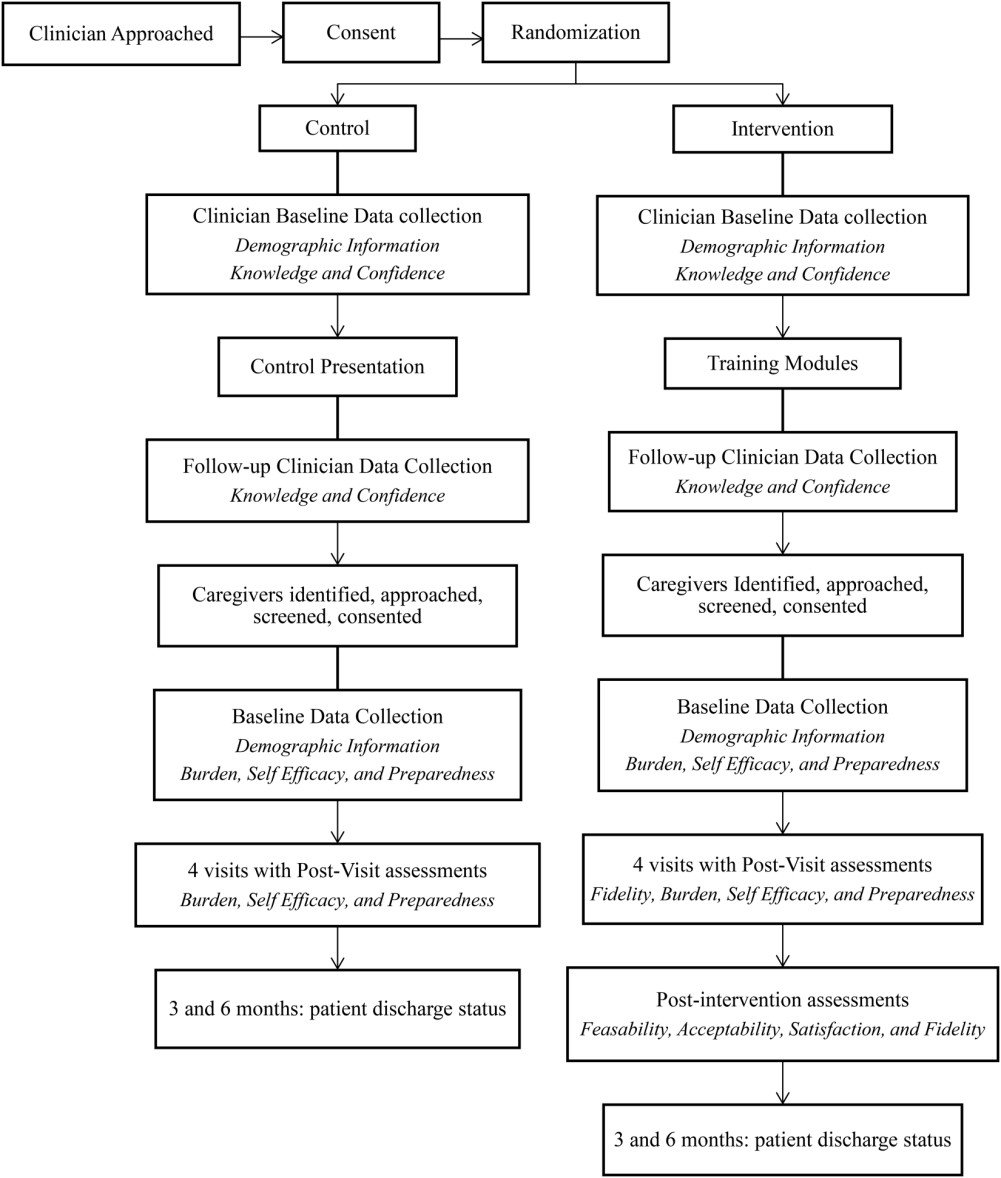
Overview of EDITH-HC Study Design. Notes. Italicized text indicates measures collected at each stage.

This pilot RCT aims to determine the feasibility and acceptability of implementing the EDITH-HC education and assessment tool in clinical practice. Additionally, it seeks to evaluate the preliminary efficacy of the intervention in reducing FCP burden compared to usual care. The study uses a non-blinded, adaptive randomization strategy to balance the number of Black and White FCP in each study arm. We plan for 24 clinicians and 96 FCPs to complete the intervention and control arms (approximately 12 clinicians and 48 FCPs in each study arm). Study enrollment began October 20, 2023 and is scheduled to conclude in March 2025. Data collection will conclude in September 2025 and data analysis in February 2026. Fig 1 provides an overview of the study design.

This protocol was approved by the VNS Health (1805421) and the Rutgers (Pro2021001817) Institutional Review Boards (IRBs) and describes the most recent version (V13, approval date July 22, 2024). No additional changes to the protocol are anticipated. This trial is registered on www.clinicaltrials.gov (NCT05719077). This study is monitored by an independent safety officer and is designed to be minimal risk. All expected and unexpected adverse events are monitored weekly and reported to the IRB’s safety officer and sponsor, as outlined in the approved data safety monitoring plan.

### Setting and participants

#### Study setting.

This study is being conducted in collaboration with VNS Health, a nonprofit healthcare provider in New York City with an average daily census of 1100 hospice patients. Intervention education modules and control presentations are delivered to clinicians over videoconference. Intervention clinicians use the assessment tool with intervention FCPs during routine home visits. Control FCPs receive usual care.

#### Eligibility criteria.

Eligible clinicians include nurses and social workers who work at VNS Health and provide care to home hospice PLwD. Eligible FCPs meet the following criteria: 1) are 18 years or older, 2) provide care to a family member or friend or other significant individual with dementia who has been enrolled in home hospice care for at least two weeks, 3) can complete data collection in English, 4) identify as Black or White, 5) provide or have provided at least eight hours of care per week for at least one month, and 6) receive a score of 15 or higher on the one-minute Animal Naming Test, a cognitive screening tool in which a person must name 15 animals in one minute. It is a well-established reliability and validity test in assessing cognitive functioning and verbal fluency [27].

#### Exclusion criteria.

Clinicians are ineligible to participate if 1) they are not a social worker or nurse or 2) they do not provide home hospice care to PLwD. FCPs are ineligible to participate if: 1) they are younger than 18 years old, 2) do not provide care to a home hospice PLwD (e.g., the patient lives in a nursing home), 3) cannot complete data collection in English, 4) do not identify as Black or White, 5) do not or have not provided at least eight hours of care per week for at least one month, or 6) receive a score below 15 on the Animal Naming Test. FCPs may also be excluded for reasons beyond those outlined. Clinicians have the discretion to exclude FCPs if they believe the caregiver would not be a good fit for the study. Additionally, exclusions may result from instances where the clinician has no established working relationship with the FCP, such as when they have not met.

### Recruitment, enrollment, and randomization

#### Clinician recruitment and enrollment.

The Principal Investigator (PI) presents the study to clinicians at regular interdisciplinary team (IDT) meetings. Clinicians are invited to express interest in the study at that time. The PI also follows up with clinicians via email and telephone to answer additional questions about the study. All questions are answered prior to obtaining oral consent.

#### FCP recruitment and enrollment.

We obtained a waiver of authorization from the IRBs at VNS Health (lead IRB) and Rutgers University for study team members employed by VNS Health to screen electronic hospice records to identify potentially eligible FCPs and contact them to tell them about the study. Once a clinician has completed their intervention education modules or control presentation and accompanying data collection, VNS Health research personnel generate a list of potentially study-eligible FCPs on that clinician’s caseload under the waiver of authorization. A trained VNS Health study staff asks clinicians to review FCPs suggested for participation and identify suitable participants (e.g., whether the individual resides in a community or nursing home setting). The VNS Health study staff contacts identified FCPs by phone using a structured telephone script to share information about the study and inquire about their interest in participating. If interested, FCPs are screened for eligibility by the VNS Health study staff. If an FCP is determined to be eligible, a study team member trained in oral consent procedures obtains informed consent telephonically. Informed consent includes a description of how participants’ data is collected, shared, and maintained to protect confidentiality.

#### Randomization.

All hospice patients work with a nurse and a social worker who are part of the same IDT, grouped by geographic area. We randomize all clinicians on an IDT to the intervention or control group when all clinicians from an IDT who are interested in participating in the study have provided consent. The first six hospice teams were randomized based on a random draw generated by the PI using Stata/MP 18.0 [28]. The remaining eight hospice teams were randomized to the intervention or control group using an adaptive randomization approach to maintain the balance between the numbers of Black and White FCPs in each study arm. FCPs are assigned to the same group as their associated clinicians. Clinicians are aimed to be matched with four or more FCPs on their caseloads, although this varies depending on clinician caseload size and composition.

### Treatment conditions

#### EDITH-HC intervention.

Clinicians in the intervention group participate in five, 30-minute educational modules consisting of four videos and one module to review the assessment tool (Table 1). Modules are delivered flexibly via videoconference—usually in 30–60-minute increments—to accommodate clinicians’ schedules. The educational videos focus on enhancing understanding and management of dementia-specific issues in end-of-life care and address supporting FCPs around issues related to: 1) eating and drinking, 2) pain assessment and management, 3) recording FCP burden in the electronic hospice record, and 4) instructions on using the intervention assessment tool. Additionally, [5] clinicians receive a separate module to be trained in the use of the intervention assessment tool. The intervention assessment tool is a one-page worksheet that helps identify and address dementia-related stressors for FCPs. The tool draws on problem-solving therapy (PST) strategies, which allow for flexible responses to the diverse challenges FCP of PLwD encounter within their unique socio-economic and cultural contexts and during end-of-life caregiving. PST approaches have demonstrated efficacy in decreasing distress, depressive symptoms, and anxiety and improving quality of life and functioning for FCP of PLwD, in end-of-life settings, and among individuals from racial and ethnic minoritized groups [29–31]. This tool aims to systematically evaluate FCP needs and guide the FCP to take concrete, realistic steps to address those needs, targeting support and resources. Clinicians use the assessment tool during routine hospice visits, providing ongoing support and education based on the identified needs.

**Table 1:** Objectives of EDITH-HC Educational Modules.

Module	Objectives
Eating and Drinking	• Provide culturally sensitive strategies for addressing emotional concerns about feeding in dementia care • Promote understanding of dementia’s impact on swallowing and appetite, supporting caregivers to set realistic expectations • Ease family distress about changes in eating habits during dementia care
Pain Assessment and Management	• Enhance the ability of caregivers to recognize non-verbal pain cues in dementia patients and tailor their care • Address caregivers’ concerns about medication use by promoting a balanced approach between non-medication and medication interventions • Foster collaboration with family members to manage pain in dementia patients, considering cultural preferences and personal care history
Needs and Strategies Video	• Enhance support for family caregivers of hospice patients living with dementia • Equip family caregivers to identify and manage stressors related to caregiving • Learn how to use a structured worksheet to guide conversations about concerns and action steps
Needs and Strategies Worksheet	• Support caregivers by reassessing and recording caregiver burden during follow-up visit • Highlight the important of ongoing caregiver assessment to ensure evolving needs are captured • Assist families in generating realistic and achievable steps to address their concerns
Electronic Health Record	• Learn how to record caregiver burden and concerns in the electronic hospice records • Understand the importance of reassessing family member concern during follow-up visits • Learn to accurately reassess and document evolving caregiver needs as a patient’s condition changes

#### Control group.

Control clinicians attend a 10-minute presentation on outcomes for hospice PLwD and their FCP. Control FCP receive usual care, which includes standard hospice services provided by VNS Health. All hospice patients receive care in accordance with the Centers for Medicare and Medicaid’s standards of care, including an in-person visit by a nurse at least every 14 days and an in-person visit by a hospice team member on at least 2 of the last 3 days of life [32,33]. Each patient receives additional visits by nurses, social workers, spiritual care counselors, and/or hospice physicians in accordance with the patient’s individualized care plan, which is evaluated on a weekly basis by the hospice team and adjusted according to the patient’s current status.

### Assessment

Clinicians complete dementia knowledge assessments at baseline and following each education module (intervention group) or the control presentation (Fig 1). FCPs complete caregiving experience questions at baseline and at follow-up for up to four routine home hospice visits with their study-enrolled clinician (Fig 1). Routine hospice visits are scheduled in accordance with individualized patient care plans. Hospice standards of care include nurse visits at least every two weeks and social work visits every month. Visits may occur more frequently, depending on patient needs. Clinicians and FCPs complete a brief fidelity assessment following each routine home hospice visit during study enrollment, up to four times for each FCP. Participants complete surveys electronically through Research Electronic Data Capture (REDCap). If they do not complete the survey online, trained study staff follow up with participants telephonically to capture data. Information about patient live discharge 3 and 6 months after the FCP completes the study will be collected from VNS Health’s electronic hospice after the last FCP concludes study activities. FCP burden, self-efficacy, preparedness, and clinician dementia knowledge measures are validated [34–38]. All data are collected and stored on REDCap [39].

### Demographic measures

Demographic information collected for participants includes age, gender, race/ethnicity, education level, years of experience (for clinicians), relationship to the patient (for FCPs), and socioeconomic status. This information will be used to describe the sample and to perform descriptive subgroup analyses to explore potential differences in intervention outcomes based on demographic characteristics. No demographic information is collected about patients.

#### Feasibility and acceptability.

The primary purpose of this study is to assess the feasibility, acceptability, and fidelity of the EDITH-HC intervention. Feasibility measures include the proportion of education modules completed by all clinicians, the proportion of clinicians completing three or more education modules, and the proportion of data collection surveys completed. Acceptability measures include the proportion of clinicians satisfied or very satisfied with the education modules and the proportion of all participants satisfied or very satisfied with the assessment worksheet. We will measure fidelity as the proportion of cases of failure to use the worksheet when appropriate and the proportion of participants using the worksheet in more than 75% of applicable visits.

### Primary outcome

We will evaluate a signal for efficacy by examining the change in FCP burden between baseline and subsequent hospice visits and compare differences in changes between the intervention and control groups. Because FCPs are relatives or loved ones of hospice patients who may die (at which point they will no longer be receiving home visits from nurses and social workers), we expect that many participants will not complete all four time points of data collection. Caregiver burden, the primary outcome, will be measured using the 12-item Zarit Burden Interview (ZBI), a validated tool commonly used in dementia care to assess caregiver burden [35,38]. The ZBI includes items that evaluate the emotional, physical, and social impacts of caregiving, with scores ranging from 0 to 48, where higher scores indicate greater burden. The intervention’s success will be indicated by a significant reduction in ZBI scores in the intervention group compared to the control group, suggesting a decrease in FCP burden due to the EDITH-HC education modules and tool.

### Secondary outcomes

Clinician knowledge about dementia care will be measured using the validated Dementia Knowledge Assessment Scale (DKAS) [34]. Responses will be scored on a scale, with higher scores indicating greater knowledge. Greater improvements in these scores from baseline to post-education modules among intervention clinicians, compared to control clinicians, will suggest the effectiveness of the educational component of the intervention.

FCP self-efficacy in managing the care of PLwD will be measured using a different scale than the clinicians, the Caregiver Self-Efficacy Scale (CSES). The CSES is a validated tool that assesses FCPs confidence in managing challenging behaviors and providing care [37]. Preparedness is measured using the Preparedness Scale, a subscale of the Family Care Inventory [36]. Increases in CSES and caregiving preparedness scores from baseline to follow-up in the intervention FCPs, compared to control FCPs, will indicate enhanced self-efficacy and preparedness among FCPs resulting from the EDITH-HC intervention.

Patient discharge status will be assessed for patients 3 and 6 months following FCP study completion using VNS Health electronic records.

### Data analysis plan

An intention-to-treat approach will be followed for all analyses proposed below, with the exception of feasibility and acceptability measures regarding intervention implementation that are only collected from intervention group participants. We will track why participants leave the study and compare those who leave to those who persist to see if attrition is random. This will help to improve design and analysis of future studies.

### Feasibility and acceptability

We will compare retention at the first follow-up (yes/no) between intervention and control groups using Fischer exact tests [40]. We will examine longitudinal study retention between intervention and control groups through all four follow-up visits using discrete Kaplan-Meier methods [41]. Otherwise, feasibility and acceptability will be assessed in the intervention group only. We will descriptively quantify feasibility and acceptability, including using proportions/means and 95% confidence intervals, as appropriate.

### Primary analysis

We plan four steps in our descriptive outcome analysis. 1) We will calculate descriptive analyses of all data by study arm, examining the balance between treatment and control groups. 2) We will establish the distribution of each of the scale outcomes at baseline by treatment arm. 3) Using t-tests of differences, we will compare FCP burden mean scores and changes with 95% confidence intervals between treatment arms at baseline and first follow-up visit. 4) We will conduct linear regression models comparing FCP burden at baseline and first follow-up visit, adjusting for baseline burden and clinician nested within the intervention arm. For primary analyses, we will use the first follow-up visit to maximize the amount of data that may be analyzed, given expected attrition due to expected high rates PLwD death after this visit during the course of the study.

### Additional analysis

In secondary analyses, we will repeat steps 3 and 4 above separately for change from baseline and second follow-up, third follow-up, and fourth follow-up visits. We will expand these analyses to combine all follow-up visits using an Analysis of Covariance (ANCOVA) approach [42]. Due to anticipated attrition, we expect the number of observations in each analysis will decrease as we progress from the first to the fourth follow-up.

We will repeat all analyses described above for clinician knowledge and caregiver self-efficacy and preparedness.

We will compare hospice live discharge status (measured dichotomously) for patients of FCPs in the intervention and control groups at 3 and 6 months post-FCP study completion using Fischer exact tests and logistical regression [40].

*Interim Analyses.* No interim analyses are planned.

*Data Sharing.* No datasets were generated or analysed during the current study.

## Results

This pilot RCT is ongoing. To date, we have enrolled 29 clinicians and 53 FCPs. We plan to conclude recruitment of FCP in March 2025. We plan to complete all data collection and data analyses by February 2026.

## Discussion

The results of this study will provide valuable insights into the feasibility, fidelity, and acceptability of the EDITH-HC intervention. By demonstrating the practicality of implementing this intervention within real-world care settings, our findings could support improved dementia-specific end-of-life care, addressing a critical gap in support for both clinicians and FCPs.

Other anticipated exploratory outcomes that are related to expanded aspects of the intervention beyond the primary outcome (reduced indications of FCP burden),such as enhanced FCP self-efficacy and preparedness and clinician knowledge, would highlight the potentially wide-ranging impact of the EDITH-HC intervention. We believe the results from this pilot RCT will provide a foundation for a multi-site, fully-powered RCT to determine intervention efficacy in a broader population.

### Limitations

There are potential limitations to consider. The specific racial demographic characteristics of our study population of English-speaking Black and White FCPs may limit the generalizability of our findings. Future adaptation and testing in additional racial and ethnic groups will be needed to minimize potential disparities in receptivity and outcomes. Additionally, while the study is designed to assess feasibility, fidelity, and acceptability, long-term impacts on FCP burden and clinician practice remain to be explored in future research. Because of the focus on feasibility and acceptability, by design, the sample size is appropriate for the study goals, but not sufficient to offset expected attrition due to patient death or to allow for analyses of additional socioeconomic or contextual factors (e.g., FCP income, employment status, cohabitation status). However, by evaluating the magnitude of attrition and comparing attritors to participants who remain in the study (i.e., through longitudinal life table and Kaplan-Meier/ proportional hazards methods), the pilot study will provide us with useful information regarding attrition to assist in study design and sample size calculations for a future, fully-powered study. Limiting participation to social workers and nurses excludes the potential benefit of the intervention to other members of the hospice team, such as spiritual care counselors and licensed practical nurses (LPNs), which future testing can address by extending to these additional hospice team members.

### Clinical implications and future directions

Should this study find positive results, the next step is a fully-powered, multi-site RCT with different hospice agencies. Should this subsequent study yield confirmatory positive results, further studies could explore the adaptability and scalability of the EDITH-HC intervention across diverse settings and populations, as well as its long-term sustainability and impact on patient outcomes. By continuing to refine and evaluate the intervention, we aim to contribute to the development of comprehensive and compassionate dementia care models that can be broadly implemented, ultimately improving the quality of life for patients, their families, and the clinicians who support them.

## Conclusion

The EDITH-HC intervention aims to enhance dementia-specific end-of-life care within hospice settings. Our study aims to establish the feasibility, fidelity, and acceptability of this intervention, providing evidence for its practical application. By reducing family care partner burden and emotional distress and improving clinician knowledge, the EDITH-HC intervention has the potential to fill a gap in current hospice care practices. If our anticipated outcomes are realized, this intervention could serve as a model for broader implementation, contributing to the development of standardized, compassionate care protocols that address the unique challenges of dementia at the end of life. Ultimately, the EDITH-HC intervention offers the potential to improve the quality of life for home hospice patients, their families, and the healthcare professionals who care for them.

## Supporting information

S1 SPIRIT Checklist SPIRIT outcomes checklist. (PDF)

S2 Oral Consent Clinician oral consent summary. (PDF)

S3 Oral Consent Family care partner oral consent summary. (PDF)

S4 Protocol EDITH-HC IRB protocol. (DOCX)

S5 Data PLOSOne human subjects research checklist. (DOCX)
