# Supplementary material for: Feasibility, acceptability, and preliminary efficacy protocol of an intervention for caregivers of hospice patients living with dementia: A pilot randomized control trial
Source: PLoS One. 2025 Nov 3;20(11):e0332360. doi: 10.1371/journal.pone.0332360 (PMC12582434; doi:10.1371/journal.pone.0332360)
Supplement: S2 Oral Consent — (PDF) [file pone.0332360.s002.pdf]

VNS Health

Consent to be Part of a Research Study

Project Title: Enhancing Dementia Instruction and Form in Home Hospice Care (EDITH-HC)
Principal Investigator: Elizabeth Luth, PhD, Rutgers
University
Co-investigator: Margaret McDonald, MSW, VNS Health

This study is supported by a grant from the National Institute on Aging.

Invitation to be Part of a Research Study

You are invited to participate in a research study. You are being invited to participate because you provide care to individuals with dementia who receive home hospice care.

Taking part in this research study is voluntary. Please take time to read this entire form and ask questions before deciding whether to take part in this research study.

What is the study about and why are we doing it?

The purpose of the study is to develop educational videos and an assessment form for home hospice nurses and social workers to learn about caring for someone with dementia or memory problems and to provide better support and care to persons with dementia and their families.

What will happen if you take part in this study?

If you agree to take part in this study, what will happen will depend on which part of the study you are assigned to.

You may be asked to listen to a presentation and answer survey questions before and after the presentations. In this case, participating in this study will take approximately 1.5 hours over several months, as it is related to family caregivers' participation and how often you meet with them.

OR

You may be asked to complete training videos and answer survey questions before and after the videos. We may also ask you to use an assessment form with 4 family members of persons with dementia on your home hospice caseload and answer survey questions about that process. You may be asked to work with more than 4 caregivers to account for clinician attrition and some clinicians being unable to be matched with 4 caregivers. We will ask you to use the form up to 4 times during your regularly scheduled visits with each of the families. In this case, participating in this study may take up to 4 hours over several months, as it is related to family caregivers' participation and how often you meet with them.

How could you benefit from this study?

Although you will not directly benefit from being in this study, others might benefit because the videos and form will help hospice nurses and social workers learn more about taking care of persons with dementia and how to better support family members who care for persons with dementia or memory problems.

What risks might result from being in this study?

The risks from being in this study are minimal. You may become uncomfortable when reviewing the

videos and form or answering questions about them. If this happens, please let the study team members know. You can choose to stop participating at any time. There is also a risk information we collect may be stolen or breached. We take steps to minimize this risk, which we explain in the next section.

How will we protect your information?

We plan to publish the results of this study. To protect your privacy, we will not include any information that could directly identify you.

We will protect the confidentiality of your research records by storing them in locked file cabinets or on secure servers that only study team members can access. Your name and any other information that can directly identify you will be stored separately from the data collected as part of the project.

It is possible that other people may need to see the information we collect about you. These people work for Rutgers University, VNS Health (formerly Visiting Nurse Service of New York), the National Institutes of Health, and government offices that are responsible for making sure the research is done safely and properly.

A description of this study will be posted on a public website, http://ClinicalTrials.gov, and summary results of this study will be posted on this website at the conclusion of the research, as required by the National Institutes of Health (NIH), the study sponsor. No information that can identify you will be posted."

What will happen to the information we collect about you after the study is over?

We will keep your research data-the answers you provide to questions we ask-to use for future research. Your name and other information that can directly identify you will be kept secure and stored separately from the research data collected as part of the project.

We may share your research data with other investigators without asking for your consent again, but it will not contain information that could directly identify you.

How will we compensate you for being part of the study?

You will receive $25 or more depending on which part of the study you are assigned to.

If you are paid more than $100, the VNS Health will collect your name, address, social security number, and payment amount. This information will be safely stored and used for income tax reporting purposes only if your total payments from the VNS Health are greater than $600 in a calendar year (January through December). If you receive more than $600 in payments from the VNS Health in a calendar year, this information will be submitted to the Internal Revenue Service (IRS) for tax reporting purposes and an extra tax form (Form 1099) will be sent to your home. If you are a VNS Health employee, your research payments are tracked separately and are not included as part of your payroll.

What are the costs to you to be part of the study?

There are no costs to you for participating in the study.

Your participation in this study is voluntary

It is totally up to you to decide to be in this research study. Participating in this study is voluntary. Even if you decide to be part of the study now, you may change your mind and stop at any time. You do not have to answer any questions you do not want to answer. If you decide to withdraw before this study is completed, we will use the data you have provided up to when you stop participating in the study.

Contact information for the study team and questions about the research

If you have questions about this research, you may contact **Dr. Elizabeth Luth, PhD at eal133@ifh.rutgers.edu or 1-848-932-5864.**

Contact information for questions about your rights as a research participant

If you have questions about your rights as a research participant, or wish to obtain information, ask questions, or discuss any concerns about this study with someone other than the researcher(s), please contact the following:

VNS Health Institutional Review Board
Phone: (212) 609-5766

What should you do if you want to be in the study?

If you want to be in the study, please provide your signature at the bottom of the page. Your signature indicates that you have read the information provided above and have decided to participate in the study.

What if you decide you no longer want to be in the study?

You may stop participating at any time after signing this form should you choose to. Your decision whether or not to participate will not impact your care or employment with Rutgers or VNS Health.
