# Supplementary material for: Feasibility, acceptability, and preliminary efficacy protocol of an intervention for caregivers of hospice patients living with dementia: A pilot randomized control trial
Source: PLoS One. 2025 Nov 3;20(11):e0332360. doi: 10.1371/journal.pone.0332360 (PMC12582434; doi:10.1371/journal.pone.0332360)
Supplement: S3 Oral Consent — (PDF) [file pone.0332360.s003.pdf]

Summary of Oral Consent to be Part of a Research Study

Project Title: Enhancing Dementia Instruction and Form in Home Hospice Care (EDITH-HC)
Principal Investigator: Elizabeth Luth, PhD, Rutgers University
Co-investigator: Margaret McDonald, MSW, VNS Health

This study is supported by a grant from the National Institute on Aging.

Invitation to be Part of a Research Study

You are invited to participate in a research study. You are being invited to participate because you provide care to individuals with dementia who receive home hospice care.

Taking part in this research study is voluntary. Please take time to read this entire form and ask questions before deciding whether to take part in this research study.

What is the study about and why are we doing it?

The purpose of this study is to test a new support program to improve care and support for persons with dementia and their families.

What will happen if you take part in this study?

If you agree to take part in this study, what will happen will depend on which part of the study you are assigned to.

You may be asked to complete a series of surveys about your experiences as a caregiver and with the hospice team up to 5 times: once when they enroll in the study, and after each of 4 visits with the hospice nurse or social worker. Each survey session will take approximately 5-10 minutes. In this case, participating in the study will take approximately 1 hour over several weeks.

OR

You may be asked to complete w worksheet with the hospice nurse or social worker during regularly scheduled visits. The worksheet will take 5-10 times to complete and we will ask you to complete it up to 4 times. You may also be asked to complete a series of surveys about your experiences with the hospice team up to 5 times: once when you enroll in the study and after each of four visits with the hospice nurse and social worker. Each survey will take 5-10 minutes. In this case, participating in the study will take approximately 2 hours over several weeks.

How could you benefit from this study?

Although you will not directly benefit from being in this study, others might benefit because the videos and form will help hospice nurses and social workers learn more about taking care of persons with dementia and how to better support family members who care for persons with dementia or memory problems.

What risks might result from being in this study?

The risks from being in this study are minimal. You may become uncomfortable when completing the worksheet or answering questions. If this happens, please let the hospice nurse or social worker or study team members know. If this happens, please let the study team members know.

You can choose to stop participating at any time. There is also a risk information we collect may be stolen or breached. We take steps to minimize this risk, which we explain in the next section.

How will we protect your information?

We plan to publish the results of this study. To protect your privacy, we will not include any information that could directly identify you.

We will protect the confidentiality of your research records by storing them in locked file cabinets or on secure servers that only study team members can access. Your name and any other information that can directly identify you will be stored separately from the data collected as part of the project.

It is possible that other people may need to see the information we collect about you. These people work for Rutgers University, VNS Health (formerly Visiting Nurse Service of New York), the National Institutes of Health, and government offices that are responsible for making sure the research is done safely and properly.

A description of this study will be posted on a public website, http://ClinicalTrials.gov, and summary results of this study will be posted on this website at the conclusion of the research, as required by the National Institutes of Health (NIH), the study sponsor. No information that can identify you will be posted.

What will happen to the information we collect about you after the study is over?

We will keep your research data-the answers you provide to questions we ask-to use for future research. Your name and other information that can directly identify you will be kept secure and stored separately from the research data collected as part of the project.

We may share your research data with other investigators without asking for your consent again, but it will not contain information that could directly identify you.

How will we compensate you for being part of the study?

You will receive between $25 each time you complete the tool and answer survey questions, up to $100.

If you are paid more than $100, the VNS Health will collect your name, address, social security number, and payment amount. This information will be safely stored and used for income tax reporting purposes only if your total payments from the VNS Health are greater than $600 in a calendar year (January through December). If you receive more than $600 in payments from the VNS Health in a calendar year, this information will be submitted to the Internal Revenue Service (IRS) for tax reporting purposes and an extra tax form (Form 1099) will be sent to your home. If you are a VNS Health employee, your research payments are tracked separately and are not included as part of your payroll.

What are the costs to you to be part of the study?

There are no costs to you for participating in the study.

Your participation in this study is voluntary

It is totally up to you to decide to be in this research study. Participating in this study is voluntary. Even if you decide to be part of the study now, you may change your mind and stop at any time. You do not have to answer any questions you do not want to answer. If you decide to withdraw before this study is completed, we will use the data you have provided up to when you stop participating in the study.

Contact information for the study team and questions about the research

If you have questions about this research, you may contact **Dr. Elizabeth Luth, PhD at eal133@ifh.rutgers.edu or 1-848-932-5864.**

Contact information for questions about your rights as a research participant

If you have questions about your rights as a research participant, or wish to obtain information, ask questions, or discuss any concerns about this study with someone other than the researcher(s), please contact the following:

VNS Health Institutional Review Board
Phone: (212) 609-5766

What should you do if you want to be in the study?

If you want to be in the study, please verbally confirm with the study team member that you have reviewed the oral consent, were able to ask questions about the study, and understood what the study is about. provide your signature at the bottom of the page. Verbally provide your agreement to participate in the study to the study team member.

What if you decide you no longer want to be in the study?

You may stop participating at any time after signing this form should you choose to. Your decision whether or not to participate will not impact your care or employment with Rutgers or VNS Health.
