# Supplementary material for: Feasibility, acceptability, and preliminary efficacy protocol of an intervention for caregivers of hospice patients living with dementia: A pilot randomized control trial
Source: PLoS One. 2025 Nov 3;20(11):e0332360. doi: 10.1371/journal.pone.0332360 (PMC12582434; doi:10.1371/journal.pone.0332360)
Supplement: S4 Protocol — (DOCX) [file pone.0332360.s004.docx]

**Enhancing Dementia Instruction and Tool in Home Hospice Care (EDITH-HC)**

**Research Protocol**

##### 1. Purpose.

This research study is intended to improve the quality of care provided to home hospice PLwD and their FCP. The purpose of this study is to develop and preliminary test an educational training program and family care partner (FCP) assessment tool for hospice nurses and social workers (clinicians)to improve clinician knowledge about end-of-life care for persons living with dementia (PLwD) and to improve support for and reduce burden among FCP of home hospice PLwD.

##### 2. Specific Aims.

The Specific Aims of the study are:

**Aim 1.** To refine dementia-focused training materials and create a goal assessment tool for home hospice clinicians to guide care and support for PLwD and FCP. Sub Aim 1. Incorporate perspectives of Black and white stakeholders (iterative feedback with engagement with 10 FCP, 5 clinicians, 5 research/content experts).

**Aim 2.** To examine feasibility and acceptability of the training and tool and revise them based on feedback. (single-armed pilot study with 1-2 hospice clinical teams (10 clinicians) and FCP (10).

**Aim 3.** To conduct a randomized pilot test to determine the feasibility and acceptability of implementing the training and tool in clinical practice and the preliminary efficacy of the training and tool compared to usual care. (two-armed randomized study with up to 14 hospice clinical teams and FCP of PLwD assigned to the clinical teams: approximately 12 clinicians and 48 FCP will be randomized to receive the intervention and approximately 12 clinicians and 48 FCP to the usual care arm. We may enroll up to 18 clinicians in each arm to allow for attrition and some clinicians not matching with 4 caregivers to reach goal of 48 FCP in each arm. Clinicians and FCP will be randomized to intervention or usual care groups by clinical team using an adaptive randomization approach that strives to maintain balance between number of Black and White caregivers in each study arm.

##### 3. Study design

This study proposes to develop and pilot test a two-part intervention, Enhancing Dementia Instruction and Tool in Home Hospice Care (EDITH-HC), that provides 1) educational videos for hospice nurses and social workers (clinicians) about dementia-specific end-of-life care and 2) an assessment tool for clinicians to use as part of their clinical practice to use with FCP to identify and address dementia-related stressors for FCP of home hospice PLwD. This study will use a mixed-methods design including intervention refinement and pilot testing. To achieve **Aim 1** we will obtain 2 rounds of structured, key stakeholder input from 10 FCP, 5 hospice clinicians, and 5 research/content experts. We will use this input to refine training materials and a tool for clinicians. The training and tool will help home hospice clinicians identify and suggest tailored strategies to address common and distressing challenges for PLwD and FCP. For **Aim 2**, we will test the training and tool with 1-2 clinical teams of 8-12 hospice clinicians and 10 Black and white FCP to determine feasibility and acceptability. We will revise the training and tool based on feedback. For **Aim 3** we will conduct a randomized pilot study to determine the feasibility and acceptability of implementing the training and tool in clinical practice compared to usual care. We will also determine preliminary efficacy of the training and tool. Outcomes include: feasibility and acceptability of the intervention, improving clinician knowledge and confidence (*secondary outcomes*), reducing FCP burden (*primary outcome*), and increasing FCP preparedness and self-efficacy, and decreasing PLwD hospice disenrollment *(secondary outcomes)*.

## **Aim 1: EDITH-HC Training and Tool Development**

**Educational Videos.** We will develop and refine 5, 5-10 minute videos, addressing topics or challenges FCPs frequently encounter in dementia caregiving at EOL, including, but not limited to, knowing what to expect as dementia progresses (Video 1), feeding as EOL approaches (Video 2), and symptom management (Video 3); and instructions on how to use the assessment tool (Video 4) and complete the care partner burden assessment embedded in the hospice’s electronic health record (EHR) (Video 5). Video content will draw upon literature review and information from prior interviews with clinicians and FCP. We will receive feedback on the videos from Black and white FCP, clinicians, and research/content experts using a modified Delphi approach incorporating user-centered design protocol (below). Videos will contain relevant information, strategies for addressing FCP challenges, and a case study to which clinicians can apply information and strategies. Providing the training in a video format will allow clinicians to complete the training at their convenience or as in-service modules, reducing burden. We will take steps to have the training count as continuing education units. Additional video topics may be developed depending on the needs of the clinicians and FCP.

**Assessment Tool.** We will develop and refine an assessment checklist adapted from a previously validated dementia caregiving burden assessment tool that focuses on and incorporates care partner burden and challenges specific to the home hospice setting. To limit burden on clinicians and FCP, the tool will provide clinicians with a series of prompts and strategies to address FCP problems identified using the assessment tool. The tool will support clinicians and FCP to identify multiple strategies for addressing issues, decide which strategy will be most effective, and devise a plan to implement the most effective strategy. The tool will be designed so that it can complement and be incorporated into standard hospice care planning. Clinicians will be trained to use the assessment tool in an educational video (previous section). We will receive feedback on the tool from Black and white FCP, clinicians, and research/content experts using a user-centered design protocol (below).

**Modified Delphi Approach with User-centered Design.** After the initial prototype development of the intervention, we will use a modified Delphi approach incorporating user-centered design process to refine the intervention. We will gather two rounds of structured feedback from key stakeholders including clinicians (5), Black (5) and white (5) FCP, and content/research experts (5). Participants will be asked to comment on the clarity and relevance of training and tool content and the feasibility of suggested strategies using structured and semi-structured interview questions. We will review and compile responses, revise intervention materials based on feedback, and request additional feedback from key informants. Informant feedback will be provided in 30-60 minute follow-up telephone, video-conferenced, or in person interviews within 4 weeks of receiving intervention materials. Clinicians and FCP will be interviewed individually and recruited from VNS Health, formerly Visiting Nurse Service of New York (VNSNY), and will be compensated $50 for each round of feedback. Research/content experts will be identified based on their expertise and compensated $250. Data collected during this process is outlined in the Appendix. Further information related to recruitment, participant inclusion/exclusion criteria, and informed consent forms are detailed in other sections of the protocol.

## **Aim 2: EDITH-HC single armed pilot study**

The aim of the single armed pilot study will be to examine feasibility and acceptability of the intervention and finalize intervention materials. Clinicians from a 1-2 VNS Health interdisciplinary teams (IDT) will be asked to view the videos and review the tool, either as part of a team meeting or on their own time. They will be asked to provide feedback after viewing each video and the tool online or via telephone or videoconference. Study staff will follow up with clinicians by phone or teleconference to collect incomplete data. FCP will review the tool with a VNS Health clinician from the IDT testing the intervention and be asked to provide feedback on it via telephone or videoconference with a member of the research team. We will obtain participants’ perceptions on feasibility of the videos and tool including utility of the individual modules, training appropriateness, and fit within organizational culture. We will evaluate the tool using a “think aloud” strategy with FCP and hospice clinicians. We will finalize the training and tool based on results from the interactive sessions. We will recruit 10 clinicians from 1-2 clinical hospice teams to test the training and tool and 10 FCP (5 Black, 5 white) to test the tool. Data collected during this process is outlined in the Appendix. Based on data, we will modify the data collection protocols and intervention before conducting the pilot RCT (Aim 3).

**Hypotheses**. >70% of participants will: 1) complete the training, 2) report the training and tool are useful, 3) increase knowledge of dementia-related challenges and care strategies, and 4) increase confidence in providing dementia-related care.

**Measurements.** All participants will be assessed on the measures listed in Table 1 (see **Appendix** for more details). Measures will be administered at baseline immediately following intervention receipt. Participants will be compensated $50 for providing feedback on the training and $50 for providing feedback on the tool. Data collected in Aim 2 will allow identification of study design issues that may not arise in Aim 1 and will provide opportunity to make refinements to the intervention before proceeding with the full pilot RCT study (Aim 3).

## **Aim 3: EDITH-HC pilot RCT**

Based on a contracted healthcare work force following the COVID-19 pandemic and resulting smaller clinical teams, we are requesting to reduce the estimated enrollment of clinicians and FCP for the pilot RCT. While we will continue to enroll as many eligible participants as possible, the range of expected participants in the following sentences represent expected “worst” and “best” case scenarios for enrollment. For Aim 3, approximately 24 clinicians and 96 Black and White FCP will be recruited from up to 14 hospice clinical teams, although we may enroll up to 18 clinicians in each arm, for up to 36 clinicians in total. The intervention will be randomized by hospice clinical team using an adaptive randomization approach that strives to maintain balance between number of Black and White caregivers in each study arm. Approximately 12 clinicians and 48 FCP will receive the intervention and 12 clinicians and 48 FCP will receive standard home hospice care, but we may enroll up to 18 in each arm to allow for clinician attrition and some clinicians not matching with 4 caregivers to reach goal of 48 FCP in each arm. The intervention will last for a few to several months, depending on the timing of FCP enrollment and routine hospice visits by enrolled clinicians. Demographic information will be collected at baseline. Feasibility and acceptability information will be collected following the training and each of three hospice clinician follow-up visits. For outcome measures, clinician knowledge and confidence in end-of-life dementia caregiving will be collected at baseline and following training completion. FCP burden, self-efficacy, and confidence will be collected at baseline and following each of three hospice clinician follow-up visits. The VNS Health and RU research study staff will coordinate to identify when study-enrolled nurses visit study-enrolled family care partners. We will use daily automated reports from the VNS Health electronic hospice record that show names of study-enrolled FCP, dates of most recent 4 visits and visit duration for study-enrolled clinicians. As needed, information will be shared with Rutgers study personnel using encrypted email or through the VNS Health approved secure MS Teams application. This information will be transferred to the study database by VNS Health or Rutgers study personnel so that participants can be contacted by phone, personalized email, or automated email (according to their stated preference), to complete relevant data collection in a timely manner. Participants will be contacted as soon as possible following, but within 2 business days, of their last clinician/family member encounter. Rutgers and VNS Health staff will work to identify the optimal time of day to contact clinicians for follow-up. Research staff will follow up with participants by phone or videoconference as necessary to complete missing data.

**Intervention.** Clinicians in the intervention group will have access to the training and assessment tool. The training may be delivered as part of a team in-service or other meeting or clinicians may access it on their own time. If the latter, they will be asked to complete the modules within 2 weeks of access being granted. They will be asked to use the assessment tool with Black and white FCP of home hospice PLwD who will be enrolled in the study by research study team members. The tool will be used during regularly scheduled visits to the patient and at up to 3 subsequent visits. Clinicians will be provided with copies of the assessment tool to complete with FCP during visits. FCP will continue to receive standard home hospice care in addition to the intervention.

**Standard Care Control.** FCP in the standard care control group will receive home hospice services as usual. These FCP will complete the same measures as participants in the intervention condition on the same timeline using identical assessment procedures.

**Hypotheses.** >70% of clinicians will 1) complete the training, 2) use the tool as intended, 3) report the training and tool are useful, and 4) report satisfaction with the training and tool. Compared to usual care: *Feasibility/acceptability:* Clinicians who complete the training and use the tool (intervention group) will have greater knowledge of dementia-related challenges in EOL care and how to manage them. Those in the intervention group will report it helps them identify individual PLwD and FCP challenges and suggest appropriate management strategies. *Primary outcome:* FCP in the intervention group will report reduced FCP burden. *Exploratory outcomes:* FCP of intervention group will experience greater self-efficacy and preparedness. PLwD of intervention group clinicians will experience increased utilization of hospice interdisciplinary team (IDT) member services and lower rates of hospice disenrollment. Exploratory analyses will examine racial disparities in FCP burden and PLwD disenrollment.

**Measurements.** All measures will be collected by study research team members via phone call or electronic survey. All participants will be assessed on the measures listed in Table 1 (see **Appendix** for more details). FCP in both arms will be compensated $25 for an initial visit followed by $25 (e.g., by ClinCard) each time they complete a follow-up survey phone call/electronic survey, up to $100. Clinicians in the intervention arm will be compensated $25 for completing the training and $25 for each FCP with whom they use the assessment tool. Our aim is to match each clinician with up to 4 caregivers, although we anticipate some will be matched with more than 4 and as such cannot state a maximum compensation amount. Clinicians in the control arm will be compensated up to $50 for completing baseline and follow up surveys assessing knowledge and confidence in addressing dementia-specific issues in end-of-life care and for completing a brief survey following routine visits with study-enrolled FCP.


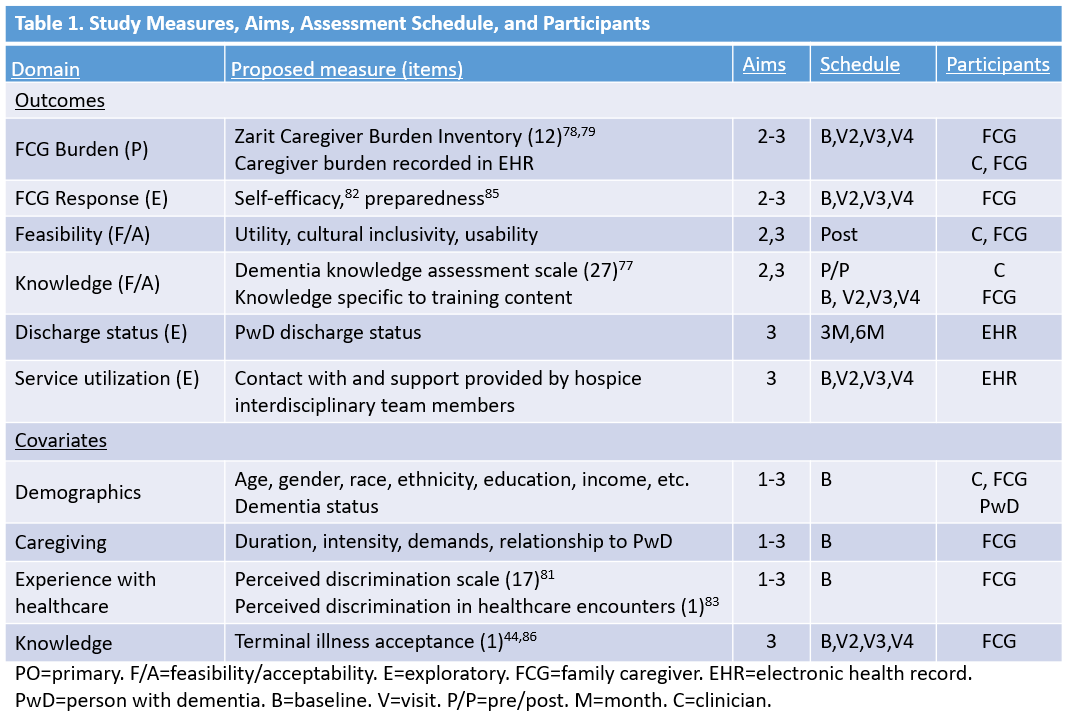


##### 4. Sample selection / Data sources

Given the disproportionate impact of dementia on Black persons, a key focus of the proposed research is to solicit input from Black and white FCP of PLwD. As such, by design we will recruit equal numbers of Black and white FCP in all stages of the study. Clinicians will not be excluded on the basis of their race or ethnicity. Based on research indicating women account for 2/3s of care partners overall, and more among Black persons, we expect 60-70% of FCP participants will be female and 30-40% male. Given women account for about ¾s of the hospice workforce, we expect 70-80% of clinician participants will be female and 20-30% male.

## **Aim 1. Intervention Development**

*Sample:* 5 Black and 5 white FCP, 5 clinicians, 5 research/content experts.

*Inclusion Criteria.* FCP participants must be identified in the medical file as the care partner, be the care partner of a recruitment site patient that is 65 years of age or older; identified as African American, black, or white; identified as having dementia; received home hospice care for a minimum of 1 week. FCP must also be 18 years of age or older, English speaking, receive a score of 15 or higher on the one-minute animal fluency test, and provide or have provided at least 8 hours of care per week for at least 1 month.

Clinicians must be a nurse, nurse practitioner, or social worker at VNS Health who provides care to older patients with dementia.

Research/content experts will possess expertise in dementia and/or end-of-life caregiving, including researchers, academics, community partners, or clinicians.

*Exclusion Criteria.* FCP are excluded if they are: under the age of 18; do not identify as African American, black, or white; do not speak English; primary relationship to recruitment site patient is employee or paid care partner; provides fewer than 8 hours of care per week; have provided care for less than 1 month; care partner of recruitment site patient that: is under 65 years of age; does not identify as African American, black, or white; does not have cognitive impairment; resides in a nursing home or long-term care facility,

Clinicians are excluded if they do not actively provide care to older adults

Research/content experts are excluded if they do not possess expertise in dementia and/or end-of-life caregiving.

## **Aim 2. One-arm Pilot Study**

*Sample.* Training: 10 clinicians. Assessment tool: 5 clinicians, 5 Black and 5 white FCP.

*Inclusion Criteria.* Same for FCP and clinicians as Aim 1. Additionally, clinicians will be part of the hospice clinical team chosen to participate in the one-arm pilot study and FCP will be part of caseload of participating clinicians.

*Exclusion Criteria.* Same for FCP and clinicians as Aim 1.

## **Aim 3. Pilot RCT**

Sample. 24 clinicians from up to 14 hospice clinical teams and 96 Black and White FCP. Approximately 12 clinicians and 48 FCP will be randomized to the intervention. Approximately 12 clinicians and 48 FCP will be randomized to usual care. We may enroll up to 18 clinicians in each arm (36 total) to allow for attrition and some clinicians not matching with 4 caregivers to reach goal of 48 FCP in each arm.

*Inclusion Criteria.* Same for FCP and clinicians as Aim 2.

*Exclusion Criteria.* Same for FCP and clinicians as Aim 2. Additionally, clinicians and FCP are excluded if they participated in Aim 2.

We are requesting a waiver of authorization to access electronic hospice records for three purposes: 1) screening to identify potential FCP participants based on hospice patient criteria described above; 2) monitoring clinician visits during the intervention to coordinate post-clinician visit data collection; and 2) identify outcome-relevant information including discharge status, number and timing of clinician visits, and clinician assessment of FCP burden.

##### 5. Procedures for recruiting study subjects.

## **Aim 1. Intervention Development**

In the first instance, FCP and clinicians will be recruited from the list of interview participants in a prior stage of this study who indicated as part of their consent process that they would like to be contacted to participate in providing feedback on the intervention. FCP and clinician participants in the prior stage met similar inclusion criteria as outlined here. Prior participants will be contacted by Rutgers or VNS Health study staff by telephone to ask if they are interested in participating in the study (Aim 1 prior participant telephone script)

If enough FCP cannot be recruited from this prior FCP participant pool, we will screen recruitment site electronic hospice records to identify additional potential FCP participants. Staff at VNS Health will review hospice enrollment records to identify potentially eligible individuals. Consistent with qualitative interviewing practices, we will use a purposeful sampling method to obtain information that is likely to be most useful in answering our research questions. We will work with healthcare providers at our recruitment sites to identify patients with a family care partner who is likely to be engaged with and provide information helpful to answering our research questions. We will recruit equal numbers of black and white FCP. They will contact these individuals to determine interest in participating in the study (Aim 1 new FCP participant telephone script), conduct an initial eligibility screening, and obtain their permission to share their name and contact information with Rutgers study personnel. VNS Health will screen potential participants for eligibility according to the criteria above. VNS Health will track the number of persons screened, number of eligibility determinations, and counts of correctly identifying care recipient, and reasons for ineligibility (number of hours of caregiving and cognitive impairment in the hospice patient). VNS Health will share a summary of screening efforts with Rutgers via email. This summary will not contain any additional or identifying information about screened individuals such as names or contact information. For interested individuals who are determined to be eligible, VNS Health will obtain informed consent and permission from the individual for VNS Health to share the individual’s contact information with Rutgers. VNS Health study personnel will provide Rutgers study personnel with contact information using encrypted email or secure file transfer. Rutgers study personnel will follow up with enrolled study participants for data collection purposes.

If enough VNS Health clinicians cannot be recruited from the prior clinician participant pool, clinicians will be recruited via email (Aim 1 new clinician participant email recruitment) and attendance at clinical team meetings to tell them about the study and gauge interest in participation. In email and at meetings, VNS Health clinicians will be provided with information on how to contact Rutgers study personnel if they are interested in participating.

Research/content experts will be identified through professional networks and/or based on their expertise in the area(s) of hospice care, dementia, and health disparities. They will be contacted by phone, mail, or email using publicly available contact information. Each individual will be approached in a personalized manner based on expertise and knowledge.

FCP and clinicians will be compensated $50 for each round of feedback on intervention materials, up to $100. Research/content experts will be compensated $250 for participation in both rounds of feedback.

## **Aim 2. Single-arm Pilot Study**

The single-arm pilot study will be conducted with 1-2 VNS Health clinical hospice teams, consisting of 8-12 nurses and social workers caring for patients in specific geographic regions of New York City. Clinical team members will be encouraged to participate in the study as part of ongoing quality improvement research. They will be asked to identify Black and white FCP of PLwD on their caseloads who they think would be willing and informative participants. VNS Health research study staff may also generate lists of patients on participating clinician caseloads and ask clinicians to review it and identify FCP who would be appropriate for the study and may be willing and informative participants. VNS Health research study staff will contact the FCP by phone to inquire about their interest in participating in the study (Aim 2 telephone script). The VNS Health research study staff will be trained to answer questions about the assessment tool and the study. If the FCP is interested, the VNS Health research study staff will screen the FCP to determine eligibility. The VNS Health research study staff will obtain the FCP’s permission to share contact information for informed consent with Rutgers research study staff so that VNS Health or Rutgers study staff can mail or email the informed consent form, obtain e-consent, call the FCP to obtain informed consent, or arrange to accompany the hospice nurse or social worker on a scheduled visit, using the method preferred by the FCP.

FCP and clinicians will be compensated $50 for participation in the single-arm pilot study.

## **Aim 3. Pilot RCT**

The 2-armed pilot RCT will be conducted with up to 14 VNS Health clinical hospice teams, consisting of 4-8 nurses and social workers. Up to 14 teams will be randomized to receive the intervention or standard care. Clinicians and FCP will be randomized to intervention or usual care groups by clinical team using an adaptive randomization approach that strives to maintain balance between number of Black and White caregivers in each study arm. We will work with VNS Health hospice leadership to select hospice teams serving similar patient populations. Hospice clinical teams will be recruited by posting the study on the VNS Health Intranet site that lists research opportunities for clinicians, email outreach, and announcements about the study during IDT team meetings. Clinical team members will be encouraged to participate in the study as part of ongoing quality improvement research. VNS Health research study staff will generate lists of potentially eligible FCP from participating clinicians’ patient caseloads and ask clinicians to review it and identify FCP who would be appropriate for the study. VNS Health research study staff will contact the FCP by phone to inquire about their interest in participating in the study (Aim 3 telephone script). The VNS Health research study staff will be trained to answer questions about the assessment tool and the study. If the FCP is interested, the VNS Health research study staff will screen the FCP to determine eligibility. The VNS Health research study staff will obtain the FCP’s permission to share contact information for informed consent with Rutgers research study staff so that VNS Health or Rutgers study staff can mail or email the informed consent form, obtain e-consent or oral consent, call the FCP to obtain informed consent, or arrange to accompany the hospice nurse or social worker on a scheduled visit, using the method preferred by the FCP.

Intervention and control group FCP will be compensated up to $100 for participation in the pilot RCT: $25 for each round of data collection including baseline and up to 3 follow-up visits. Intervention group clinicians will be compensated $25 for completing the training and $25 for each FCP that they work with during the study and for whom they respond to survey questions. Our goal is to match each clinician with up to 4 caregivers, but we anticipate some will be matched with more than 4 and as such, cannot state a maximum compensation amount. Control group clinicians will be compensated up to $50: $25 for completing the baseline and post-presentation assessments and $25 for completing the post-visit assessments.

## **Recruitment Documents**

## **Aim 1 – Prior Participant telephone script recruitment (clinicians and FCP)**

Good [morning/afternoon/evening]. May name is [insert name]. I am calling from Visiting Nurse Service of New York. May I please speak with Mrs./Ms./Mr. [care partner last name]?

I am calling to thank you again for participating in the interview for our research study for family members or close others who provide or recently provided assistance to a loved one with memory problems or dementia.

At the time of the interview, you indicated you were willing to be contacted regarding the second part of the study, and that is why I am calling today. We are asking people who completed an interview if they are interested in providing feedback on the materials we developed based on the information from your and others’ interviews.

Would you like to hear more about the second part of the study?

*If YES, continue*.

*If NO, thank the person for their time.*

We developed educational videos and an assessment tool and are seeking input to improve the videos and assessment tool to improve them for future use.

The study will be conducted by VNS Health and Rutgers University at VNS Health headquarters, {during IDT meetings}, by telephone, or by videoconference at your convenience and will last 2-3 hours over two sessions. You will be shown a prototype of the materials and a research assistant will ask you for feedback. You will be compensated for each session with a $50 gift card, up to $100.

Do you have any questions?

*Answer all questions.*

Are you interested in participating?

*If YES, confirm contact information for mailing/emailing informed consent and schedule time to review it*.

*If NO, thank the person for their time.*

NOTE: Language in {} will be included for clinicians only.

## **Aim 1 – Prior participant email/letter recruitment (clinicians and FCP)**

Subject: Research Study Part 2

Dear [Colleague / name of participant]:

Thank you again for participating in the interview portion of our research study at Weill Cornell Medicine for family members or close others who provide or recently provided assistance to a loved one with memory problems, or dementia. Based on the information provided in that study, we are developing an intervention for hospice clinicians to improve support for families of persons with dementia or memory problems.

At the time of the interview, you indicated you were willing to be contacted regarding the second part of the study, which is why we are reaching out to you. We are asking people who completed an interview if they are interested in providing feedback on the materials we developed based on the information from your and others’ interviews. We developed educational videos and an assessment tool and are seeking input to improve the videos and assessment tool to improve them for future use.

The study will be conducted by VNS Health and Rutgers University at VNS Health headquarters, {during IDT meetings}, by telephone, or by videoconference at your convenience and will last 2-3 hours over two sessions. You will be shown a prototype of the materials and a research assistant will ask you for feedback. You will be compensated for each session with a $50 gift card, up to $100. If you are interested in participating, please reply to this email, or email or call [research assistant] at [email, phone number].

This study is confidential and participation is entirely voluntary. Your decision whether or not to participate will not affect your relationship {or employment} with VNS Health or Rutgers. Your response is an invaluable part of our research and will help us better understand how to improve care for persons with dementia and their families. If you have any questions or comments about this work, please call or email at [phone number] [eal133@ifh.rutgers.edu](mailto:eal133@ifh.rutgers.edu)

Thank you for your consideration.

Sincerely,

Elizabeth Luth, PhD

Study title: Enhancing Dementia Instruction and Tool in home Hospice Care (EDITH-HC)

IRB# [IRB #]

NOTE: Language in {} will be included for clinicians only.

## **Aim 1 – New Clinician Participant Email recruitment (clinicians)**

Subject: NIH-funded study participation request – Enhancing Dementia Instruction and Tool in Home Hospice Care (EDITH-HC).

Dear [Colleague / name of participant]:

We are contacting you to ask for your participation in an NIH-funded research study to obtain feedback on an intervention to improve care for home hospice patients with dementia and their care partners. The intervention is called Enhancing Dementia Instruction and Tool in home Hospice Care (EDITH-HC). It consists of educational videos and an assessment tool. We are recruiting home hospice nurses and social workers to obtain feedback on the intervention so we can improve it for future implementation.

The study will be conducted at VNS Health headquarters, during IDT meetings, by telephone or by videoconference at your convenience and will last 2-3 hours over two sessions. You will be shown a prototype of the intervention and a research assistant will ask for your feedback. You will be compensated for each session with a $50 gift card, up to $100. If you are interested in participating, please reply to this email.

This study is confidential and participation is entirely voluntary. Your decision whether or not to participate will not affect your employment at VNS Health in any way. Your response is an invaluable part of our research and will help us better understand how to improve care for home hospice patients with dementia and care partners. If you have any questions or comments about this work, please email at eal133@ifh.rutgers.edu

Thank you in advance for your consideration.

Sincerely,

Elizabeth Luth, PhD

## **Aim 1 – New Participant telephone script recruitment (clinicians and FCP)**

Good [*morning/afternoon/evening*]. May name is [*insert name*]. I am calling from Visiting Nurse Service of New York. May I please speak with *Mrs./Ms./Mr. [care partner last name]*?

I am calling to tell you about a project that researchers at Rutgers University and VNS Health are conducting and to see if you would be interested in participating.

Would you like to hear more about the study?

*If YES, continue*.

*If NO, thank the person for their time.*

The project involves providing feedback on educational videos and an assessment tool to improve care for persons with dementia and their families. If you agree to participate, we will send you the materials and conduct a videoconference or phone call with you to ask you questions and get feedback. We will then revise the materials based on all participant feedback and ask you to review the revised videos and assessment tool in a similar videoconference of phone call. Reviewing the materials and providing feedback will take approximately 2-3 hours and you will receive a $50 gift card each time you provide feedback, up to $100.

Do you have any questions?

*Answer all questions.*

Are you interested in participating?

*If YES, proceed to screening for family care partners*.

*If NO, thank the person for their time.*

## **Aim 2 – FCP telephone script recruitment**

Good [*morning/afternoon/evening*]. May name is [*insert name*]. I am calling from VNS Health. May I please speak with *Mrs./Ms./Mr. [care partner last name]*?

I am calling to tell you about a project that researchers at Rutgers University and VNS Health are conducting and to see if you would be interested in participating. We understand that this may be a difficult time for you and your participation is entirely voluntary.

Would you like to hear more about the study?

*If YES, continue*.

*If NO, thank the person for their time.*

The purpose of this study is to test a new intervention to improve care and support for persons with dementia and their families. The intervention involves working with the hospice nurse or social worker to complete and assessment tool. You will receive this intervention in addition to the care you are currently receiving.

If you agree to participate, the hospice nurse or social worker will complete the assessment tool with you during one of your regularly scheduled visits. The assessment tool will take approximately 10-15 minutes to complete. In addition to completing the tool, we will call you by phone to ask some survey questions, which will take approximately 30 minutes. You will receive a $50 gift card for your time completing the assessment tool and answering the questions.

Do you have any questions?

*Answer all questions.*

Are you interested in participating?

*If YES, proceed to screening*.

*If NO, thank the person for their time.*

## **Aim 3 – FCP telephone script recruitment**

Good [*morning/afternoon/evening*]. My name is [*insert name*]. I am calling from Visiting Nurse Service of New York. May I please speak with *Mrs./Ms./Mr. [care partner last name]*?

I am calling to tell you about a project that researchers at Rutgers University and VNS Health are conducting and to see if you would be interested in participating. We understand that this may be a difficult time for you and your participation is entirely voluntary.

Would you like to hear more about the study?

*If YES, continue*.

*If NO, thank the person for their time.*

The purpose of this study is to test a new intervention to improve care and support for persons with dementia and their families. The intervention involves working with the hospice nurse or social worker to complete and assessment tool. You will receive this intervention in addition to the care you are currently receiving.

If you agree to participate, the hospice nurse or social worker will complete the assessment tool with you their regularly scheduled visits. The assessment tool will take approximately 5-10 minutes to complete and we will ask you to complete it up to 4 times. In addition, each time you complete the tool, we will call you by phone to ask some survey questions, which will take approximately 15 minutes. You will receive a $25 gift card each time you complete the assessment tool and answer the survey questions, up to $100.

Do you have any questions?

*Answer all questions.*

Are you interested in participating?

*If YES, proceed to screening*.

*If NO, thank the person for their time.*

## **FCP screening tool (Aim 1 new participants, Aims 2-3)**

1. Do you provide help or support to a family member or loved one who is/was enrolled in VNS Health hospice care? (Must have a loved one or family member enrolled in VNS Health hospice)
2. Does/Did your family member/[insert relationship] have memory problems or dementia? (Must have memory problems or dementia)
3. Are you over 18 years old? (Must be 18 years or older)
4. Do you identify as African American, black, white, or Caucasian? (Must identify as one of these categories. May identify as both or additional races/ethnicities.)
5. Does/Did your family member/[insert relationship] live in a nursing home or other long-term care facility [when they died]? (Must receive/have received home hospice care)
6. For how long [have you been caring/did you care] for your family member/[insert relationship]? (Must provide care for at least 1 month)
7. About how many hours a week [do/did] you spend caring for your family member/[insert relationship]? (Must provide at least 8 hours of care/week)
8. Please name as many animals as you can in one minute. (Write down every animal mentioned. Must name 15 different animals)

Note. Past tense language allows for inclusion of bereaved care partners in Aim 1. For Aims 2 and 3, all participants must be care partners of current hospice patients.

##### 6. Consent forms and additional PHI authorizations.

For all three Aims, and prior to participating in any study activities, all FCP, clinician, and research/content expert participants will provide informed consent. Informed consent will be obtained by study personnel in person, by telephone, or using an electronic consent process. The format of informed consent will be based on the study participant’s stated preference. Study participants will sign the approved informed consent or e-consent form and be provided with a copy of the signed document.

For Aim 3 we will also have an oral consent option and are requesting an alteration of written consent in order to provide oral consent as an alternative to written or e-consent, should this be the participant’s preferred method of providing consent. We are adding oral consent as an option due to feedback from previous aims. We found that one reason that hospice caregivers were not participating was that many of them are very busy. We hope that by offering an oral consent option, this will allow more caregivers to participate by reducing the burden of study participation and include individuals in the study who may not have access to technology to provide e-consent. Please see attached oral consent script, oral consent form, and changes in the protocol to reflect this addition. After obtaining oral consent over the phone, we will mail or email the consent form to the caregiver to have as a copy. We will still maintain written consent as an option for caregivers who want to meet in-person.

Informed consent will be conducted by study personnel trained in consent procedures. Study personnel will review the study, allowing the participant to ask questions at any time during the discussion. The main points addressed by study personnel include explaining that participation is voluntary, why the study is being done, study procedures, potential risks and benefits of participation, and how participant confidentiality will be protected. Electronic copies of informed consent documents will be stored on a secure server that only authorized study personnel are credentialed to access. E-consent forms will be collected, and oral consent will be tracked and stored in a secure, HIPAA compliant database such as REDCap, that only authorized study personnel are credentialed to access.

Copies of consent forms for Aims 1-3 and the oral consent procedures for Aim 3 are included in the application package. An example of the electronic informed consent form can be found here:

<https://redcap.rwjms.rutgers.edu/surveys/?s=XMDTAPVMxS>

A modification has been made as of May 2024 to increase the number of family caregivers with whom clinicians can be matched to offset caregiver attrition and maximize participation among clinicians that may have an opportunity to match with more than 4 family caregivers. Study staff will return to currently enrolled clinicians and ask if they are willing to match with more than 4 caregivers if such matches are available, collecting verbal consent in the process.

##### 7. Request for Waiver of Authorization

In addition to obtaining consent from FCP, clinician and research/content expert participants, we are requesting a waiver of consent and HIPAA authorization to access electronic hospice records for the following reasons.

**Planning**. We will extract information about the care partner burden field and the individuals for whom it is completed in the electronic hospice record, including care partner burden scores, dates recorded, patient gender, race, ethnicity, age, primary and comorbid diagnoses, dates of enrollment and discharge, discharge status, care partner relationship to patient, and clinician recording burden rating. We will also extract similar information for fields relevant to the content of the educational training videos (e.g. care partner education, prognostic understanding, etc.). Analyzing this information will allow us to tailor educational videos and training materials for clinicians. As this extraction will involve several thousand records, a majority for deceased individuals, it is not possible to obtain this information without a wavier of authorization.

**Aims 1-3.** We request the waiver to view electronic hospice records of home hospice patients in order to identify FCP who are potentially eligible to participate in all three aims of the study. It is not possible to identify potentially eligible FCP without the waiver of authorization.

**Aims 2-3.** We request the waiver to view electronic hospice records of home hospice patients of study-enrolled FCP to obtain the dates on which clinicians visit these patients. We require this information in order to know when to follow up with FCP to assess intervention fidelity and collect outcomes data (burden, self-efficacy, etc.) and when to follow up with clinicians to assess intervention fidelity. Because all patients have dementia, it is not possible to obtain information about hospice clinician visit timing from patients directly.

**Aim 3.** We request the waiver to extract outcome-related information from electronic health records of home hospice patients whose FCP are enrolled in the pilot RCT (Aim 3). We will extract his information 3 and 6 months following FCP participation in the pilot RCT and so anticipate that a majority of the hospice patients for whom the information is extracted will be deceased. For these individuals, we will extract information regarding their hospice enrollment and services, including: gender, race, ethnicity, age, dates of enrollment and discharge, discharge status, care partner burden and dates recorded, care partner relationship to patient, dates and duration of hospice interdisciplinary team visits, and the clinician who made the visit.

A Request for Waiver of Authorization Form is included in the application.

##### 8. Data to be employed

Please see Table 1 (above) and Appendix for data collection instruments at the end of the document. Data collection procedures will be conducted by trained research staff and done in-home, at VNS Health headquarters, at Rutgers University, by phone, by videoconference or online surveys as outlined in the protocol above. Data will be collected by research staff or online questionnaires and entered into and stored in a HIPAA compliant database such as REDCap.

##### 9. Analytic Methods.

**Feasibility/Acceptability.** We will assess feasibility and acceptability using accrual rates, attrition rates, adherence to the study protocols, use of the intervention (e.g. educational video views, training completion, use of assessment tool), and participant evaluations of perceived utility and acceptability of the intervention using a feasibility questionnaire (Appendix).

**Qualitative analysis.** We believe sample sizes will be sufficient to obtain thematic saturation in qualitative analyses. If needed, we will continue to collect data at all stages until achieving thematic saturation. Data coding and analysis will be informed by a responsive model in which blocks of information are combined based on the theme they represent. Trained raters will independently review the transcripts and identify passages that include suggestions for inclusion and/or modifications to the intervention. Raters will then organize these passages into categories reflecting single themes, discuss these themes, and revise themes until consensus is reached. Identified themes will inform modifications to the intervention. Additionally: **Aim 1**. A study team member will take notes during stakeholder interviews. Relevant notes and written feedback from stakeholders will be incorporated into training and tool refinement. **Aim 2**. Participant responses to questions about intervention components will be recorded in a secure database (e.g. REDCap). Responses will be systematically analyzed by research team members, under the supervision of the Principal Investigator, to identify items for revising and finalizing the training and tool and implementation processes. When participants provide responses by telephone or videoconference, we will record responses and transcribe them into REDCap and analyze them for themes.

**Quantitative Analysis.** We will calculate descriptive statistics to characterize the sample of participants in each aim. **Aim 3.** We will calculate changes in participant outcomes by comparing individual responses to questions at baseline and various points in time after exposure to the training and tool. To establish preliminary efficacy, we use a continuous measure of FCP burden using the 12-item Zarit Burden Interview (ZBI). The primary analysis will be a full information mixed model approach. We base primary power calculations on a more conservative two-group comparison of endpoint means (differences in means) with possible attrition. We assumed σ (variance)=9.8 (for the ZBI), α=0.05 R (reliability)=0.9 and g=2 groups (intervention and usual care). We estimated the variance inflation factor from the design effect of clustering within clinician: Vif=1+(Nc-1)Icc=1.09 (with cluster size, Nc=4 patients per provider) and intracluster correlation coefficient (Icc=0.03). Assumptions are based on earlier care partner studies in racial/ethnic minority samples. Assuming power of 0.80, with 80 per group, we would be able to detect a moderate effect size (Cohen’s d89=0.49), equivalent to ~4.80 points on the ZBI. We also examined sample size requirements for the detection of other endpoint differences: 4.0, 4.5, and 5 points on the ZBI. We examined different scenarios regarding correlations between baseline and follow-up outcome measures. We modified the formula from Fleiss (p 4-5) to include different scenarios related to correlations between the two waves of data: n*= [4(1-ρ)(σ2)(Zα/2+Zβ)2] / δ2 adjusting for unreliability: n=(n*Vif) / R.

The power calculation is based on a sample of 48 care partners and 4 care partners per clinician, and ρ (correlations between repeat measures of each person in waves of data)=0.5, 0.6 and 0.7, the resulting estimates of minimal detectable intervention arm differences are δ=6.21, 5.56, and 4.81 for ZBI, thus demonstrating that a medium effect size (Cohen’s d=0.49 to 0.63) could be detected with this sample size. As the study progresses, actual enrollment may change and analyses will be adjusted accordingly.

We believe FCP burden will be the primary outcome for this intervention. However, as this is a pilot trial, we want to ensure we identify the appropriate outcome for a fully-powered RCT, and so will also collect information about changes in FCP self-efficacy and preparedness and clinician knowledge and confidence in EOL dementia care. The purpose of the pilot testing is to determine feasibility of implementing the training and tool in a clinical setting and establish their preliminary efficacy in reducing FCP burden, collect normative data, and identify the appropriate outcome so that a fully-powered RCT can be conducted in future research (R01). In order to lay the groundwork for a future pragmatic trial of the intervention, we will also collect exploratory data existing in the hospice EHR including care partner burden, information related to educational videos content (e.g. care partner education, prognostic understanding, etc.), discharge status, and patterns of IDT visits to PLwD.

##### 10. Statement of the risks/benefits for the study subjects.

This is a minimal risk study to develop, pilot test the feasibility, acceptability, and preliminary efficacy of an intervention consisting of educational videos and a care partner assessment tool.

**Aim 1.** Intervention Development. We do not anticipate that participants will benefit directly from providing feedback on the intervention materials. Risks to study participants are minimal and may include FCP becoming distressed when reviewing material about end-of-life care and potential stressors.

**Aim 2.** Single Arm Pilot. The purpose of this aim is to test feasibility and acceptability of implementing the intervention within a hospice clinical team and a small number of family care partners. We do not anticipate participants will benefit from their participation. Clinicians may improve their knowledge of dementia caregiving at end of life and their confidence in supporting patients living with dementia and their FCP. FCP may feel reduced burden and/or increased self-efficacy and preparedness after pilot testing the assessment tool. Risks to study participants are minimal and may include becoming distressed when identifying stress or issues related to their family caregiving. The intervention is designed to fall within the normal scope of nurses’ and social workers’ jobs, and so in most cases, they will be equipped to address FCP distress. Clinicians will follow normal VNS Health procedures should a FCP become distressed.

**Aim 3.** Pilot RCT. This aim pilot tests feasibility and acceptability of implementing the intervention on a larger scale. There are no anticipated benefits for control group participants. In the intervention group, clinicians may improve their knowledge of end-of-life dementia caregiving and confidence in supporting patients and their FCP. FCP may feel reduced burden and/or increased self-efficacy and preparedness after using the assessment tool. For both groups, FCP may feel burdened answering survey questions after clinician visits. We have kept c burden to a minimum to reduce this risk. There are no anticipated additional risks for control group participants. Intervention group FCP participants may feel distressed when identifying stress or issues related to their family caregiving. The intervention is designed to fall within the normal scope of nurses’ and social workers’ jobs, and so in most cases, they will be equipped to address FCP distress. Clinicians will follow normal VNS Health procedures should a FCP become distressed. Additional steps to minimize risk and address adverse events are outlined in the Data Safety Monitoring Plan included in this application.

**Aims 1-3.** All study subjects will be free to refuse to answer any questions or to discontinue study participation at any time. As always, there is the possibility that participant confidentiality will be breached. We will take steps to minimize this possibility and protect participant confidentiality as outlined in the following section.

**Aims 2-3**. FCP participants may become emotional or upset when completing the tool with clinicians because they are being asked to think about and address something that is causing them stress or worry. As VNS Health hospice clinicians routinely discuss and address with FCP sources of their stress, worry, and anxiety, they are well equipped to handle the types of emotions that may arise as part of completing the tool. In these situations, they will follow VNS Health standard procedures: nurses will offer a referral to the VNS Health social worker and/or spiritual care counselor. Social workers will use their training to help FCP cope with their emotions and offer referrals to the VNS Health spiritual care counselor and/or external resources as appropriate.

##### 11. Privacy / Confidentiality Protections.

Interview and focus group data will be transcribed and imported into qualitative data analysis software for analysis (e.g. nVivo, Dedoose). All identifying information will be removed from transcriptions. Study IDs will be substituted for names. All participant and quantifiable study data will be stored in a HIPAA compliant manner, such as a password-protected electronic database (REDCap) and/or in a file on a secure Rutgers server accessible only by the PI and key study personnel. Collected data will only be identified via a study ID that will serve as a link to identifiable information in a separate file on a password-protected, secure, Rutgers server or in a separate part of the password-protected electronic database (REDCap). Data will be analyzed in aggregate only and no identities will be revealed. For qualitative reporting, participants may be identified by pseudonyms or general descriptors (e.g. “daughter care partner,” “physician”).

##### 12. Data Use Agreement.

A subaward agreement outlines the scope of work and research data to be shared between VNS Health and Rutgers (see “Subaward Agreement.”)

##### 13. Rutgers IRB Approval.

Rutgers IRB approval is pending and will be submitted to the VNS Health IRB prior to data collection or sharing begins.

##### 14. Reporting.

The PI, Dr. Luth, will adhere to VNS Health IRB reporting requirements, including submitting a study closure report within 60 days of the end of primary data collection. The PI will also submit a summary of main project findings when available.

##### APPENDIX: Data Collection Tools

# Aims 1-3. Demographic Questions (Baseline)

1. What is your date of birth?
2. What is your gender?

□ Male

□ Female

□ Other

1. What is your race/ethnicity?

□ White

□ Black or African American

□ Asian

□ American Indian or Alaska Native

□ Native Hawaiian or other Pacific Islander

□ Multi-racial (specify)

□ Other, please specify

□ Don’t know/unsure

□ Prefer not to answer

1. Are you of Hispanic, Latino/a, or Spanish origin?

□ Yes

□ No

□ Prefer not to answer

□ Don’t know/unsure

1. (If YES to Q4). What is your family’s country of origin? [if multiple indicated, ask person to select the one with which he/she most strongly identifies]

□ US

□ Puerto Rico

□ Mexico

□ Dominican Republic

□ Cuba

□ Other (specify):

□ Don’t know/unsure

1. What is your religion?

□ Catholic

□ Baptist

□ Protestant

□ Jewish

□ Muslim

□ Pentecostal

□ Atheist

□ Agnostic

□ Other (specify)

□ Prefer not to answer

□ Don’t know/unsure

Other:________________________________

1. What was the highest education level you completed?

□ Some elementary school

□ Completed elementary school

□ Some high school

□ High school or GED

□ Some college

□ College degree

□ Post-graduate but no degree

□ Graduate degree

□ Prefer not to answer

□ Don’t know/unsure

1. Are you currently employed?

□ Yes

□ No

□ Prefer not to answer

□ Don’t know/unsure

(If no, skip to question 9)

1. If you are employed, is it:

□ Part-time

□ Full-time

1. Are you retired?

□ Yes

□ No

□ Prefer not to answer

□ Don’t know/unsure

1. What is/was your occupation?

11a. How long have you been in this occupation? _______________

1. We ask about participants’ socioeconomic backgrounds because we think it is important to understand how people from different backgrounds differ in their experiences with dementia and hospice. Would you mind telling me the annual, combined income range for all family members in your household?

What is your annual income, before taxes?

□ Less than $25,000

□ $25,000 to $49,999

□ $50,000 to $74,999

□ $75,000 to $149,999

□ $150,000 or more

□ Prefer not to answer

□ Don’t know/unsure

1. What is your marital status?

□ Single

□ Married

□ A member of an unmarried couple

□ Divorced

□ Separated

□ Never married

□ Widowed

□ Prefer not to answer

□ Don’t know/unsure

1. Do you have children?

□ Yes

□ No

□ Prefer not to answer

□ Don’t know/unsure

15. (If YES to Q14). How many children?

1. What is your role?

- Family Caregiver
- Clinician
- Other (specify)

## ***Additional question for clinicians***

1. What is your occupation? (collected as part of initial screening)
   - - 1. Nurse practitioner
       2. Nurse
       3. social worker
       4. other (please specify)
2. How long have you been working in hospice care?
3. How often do you typically see a patient during their hospice stay?
4. One time
5. A few times, as needed
6. Monthly
7. Every two weeks
8. Weekly, or more often

***Additional questions for FCP:***

Now I would like to ask you some questions about your experience providing help and support to your loved one.

1. What is your relationship to your family member? I am his/her:

□ Spouse / Partner

□ Daughter

□ Son

□ Daughter-in-law

□ Son-in-law

□ Sister

□ Brother

□ Sister-in-law

□ Brother-in-law

□ Granddaughter

□ Grandson

□ Other(specify)

1. For how long have you been providing help or care to your family member?
2. On average, how many hours a week do you help your family member?

□ < 8 hours

□ 8 to <15 hours

□ 15 to <30 hours

□ 30 hours or more

□ 24 hours a day, 7 days a week

1. In the past two weeks, how often have you helped your family member with self-care tasks, including dressing, bathing, toileting, eating, or getting out of bed/around the home?

□ Daily/Almost every day

□ 2-3 times a week

□ 2-3 times in the last two weeks

□ I have not helped with any of these

1. In the past two weeks, how often have you helped your family member with activities including cooking, cleaning, providing transportation, laundry, or managing finances?

□ Daily/Almost every day

□ 2-3 times a week

□ 2-3 times in the last two weeks

□ I have not helped with any of these

1. In the past two weeks, how often have you helped your family member manage their health including managing or giving medications or coordinating with healthcare providers such as hospice or doctor’s offices?

□ Daily/Almost every day

□ 2-3 times a week

□ 2-3 times in the last two weeks

□ I have not helped with any of these

# Aim 1. Think Aloud Interview (Round 1 feedback)

Thank you again for your time and help with this project. The goal of the project is to improve educational videos and an assessment tool to improve home hospice care and support for persons with dementia and their families. The videos are designed to provide information to hospice nurses and social workers. The assessment tool is designed to be completed in conversation with hospice nurses or social workers and families of persons with dementia. We would like to get your feedback on the videos and assessment tool and will use your feedback to improve them. I will ask you questions about each as we review them. Do you have any questions before we begin?

Videos

[Ask questions for each video]

1. I want to understand what you think about the video. Watch it and think about the information it contains. Tell me things you might be thinking as you watch.

What part of the video was most helpful?

Why do you think this is the most helpful?

1. Describe the message of the video in your own words.

Was there any part of the message that was confusing to you?

Are there parts of the message that you think would be helpful to a family member of a person with dementia?

1. Do you have any additional thoughts or suggestions for improving this video? This might include additional information we should include, topics you would like to see addressed or removed?
2. What do you think is the most important thing for hospice nurses and social workers to understand about the topic addressed in the video?

Do you think that was covered in the video? What additional information should we include?

The next set of questions ask about particular parts of the video.

1. Did you have difficulty understanding the narrator?

Do you have suggestions for making it easier to understand the narrator?

1. Did you have difficulty understanding the slides?

Do you have suggestions for making it easier to understand the slides?

1. Is the amount if information in the video appropriate? For example, is there too much or too little information in the video?
2. How often do you think you would watch these videos? For example, once? A few times? A different timeline?

Assessment Tool

1. What part of the assessment was most helpful?

Why do you think this is the most helpful?

1. Describe the purpose of the assessment tool in your own words.

Was there any part of the assessment tool that was confusing to you?

Are there parts of the assessment tool that you think would be helpful to a family member of a person with dementia?

1. Do you have any additional thoughts or suggestions for improving this assessment tool? This might include additional information we should include, topics you would like to see addressed or removed?

The next set of questions ask about particular parts of the assessment tool.

1. Did you have difficulty understanding the assessment tool?

Do you have suggestions for making it easier to understand the assessment tool?

1. Did you have difficulty understanding the slides?

Do you have suggestions for making it easier to understand the slides?

1. Is the amount if information in the assessment tool appropriate? For example, is there too much or too little information in the assessment tool?
2. How often do you think you would use the assessment tool? For example, once? A few times? Every time a hospice nurse or social worker visits? A different timeline?
3. What do you think is the most important thing for hospice nurses and social workers to ask about in the assessment tool?

Do you think that was covered in the video? What additional information should we include?

1. Would you be willing to use the assessment tool during a phone call or telehealth visit?

What are the benefits of using the assessment tool over the phone? During a telehealth visit?

What are the drawbacks of using the assessment tool over the phone? During a telehealth visit?

Do you have suggestions for a better method for using the assessment tool?

1. How much time should it take to complete the assessment tool?

**Aim 1. Think Aloud Questionnaire for Round 2 of Data Collection**

[Ask questions for each video]

1. We have some questions about the language used in the [video/tool].
   1. Were there words that you found confusing or that you didn’t understand?
   2. Were there words you found off-putting, or that you would not use to describe your experience?
   3. What words would you suggest in place of the ones we used?

[Prompt for reaction to the following words: progression, addiction, caregiving, burden, strain, stressor, strategy, advantage, disadvantage, etc.]

1. Are there changes you would suggest to make the [video/tool] more visually pleasing?

[Prompt for reaction to: slide color, font type, font size, visual aids (icons, photos, graphics)]

1. Are there changes you would suggest to make the [video/tool] more reflective of the hospice experience?
2. Are there changes you would suggest to make the information provided in the [video/tool] better organized or easier to follow?
3. [For relevant video] What did you think about the closed captioning? [Prompt: Did you find it helpful or distracting? Is this something you would like to see for all of the videos?]
4. Do you have other suggestions to improve the [video/tool]?
5. Is there anything you would suggest we definitely keep or do not change in the [video/tool]?

Additional questions for Assessment Tool:

1. How helpful was the video in explaining how to use the tool?
2. Do you think it is necessary to watch the video in order to use the tool correctly? Why or why not?
3. How confident would you be using the tool without having seen the video? Why do you think that?

## **Aims 2 and 3. Pre/Post Video Questions.**

Clinicians are asked these questions as part of baseline data collection upon enrollment. Relevant questions are also asked after viewing each video.

Instructions. Except where indicated, all questions are asked at study enrollment as part of baseline data collection and again after each video or upon completion of all training modules.

All questions regarding confidence are answered on a 1 (not at all confident) to 10 (extremely confident) point scale.

Confidence Questions are asked all at once pre-video and immediately following each relevant video.

(responses: 1= not confident at all, 2, 3, 4, 5=somewhat confident, 6, 7, 8, 9, 10=very confident).

1. How confident are you in providing dementia-related care to hospice patients?
2. How confident are you in providing support to family members of hospice patients living with dementia?

Nutrition video:

In thinking about your home hospice patients living with dementia and their family members:

1. How confident do you feel **assessing** a patient’s eating, drinking, and nutrition needs?
2. How confident do you feel **assessing family member education needs** with respect to eating and drinking?
3. How confident do you feel **talking with families** about a patient’s eating, drinking and nutrition when problems arise?
4. How confident do you feel **providing family members with information or education** about eating and drinking?
5. How confident do you feel **providing family members with concrete strategies or steps** they can implement to address their loved one’s eating and drinking?
6. How confident do you feel **talking with families about feeding tubes**?
7. How confident do you feel **providing families with education or information** about feeding tubes?

Pain video

In thinking about your home hospice patients living with dementia and their family members:

Pain Assessment

1. How confident do you feel **assessing** a patient’s pain?
2. How confident do you feel **assessing family member education** **needs** with respect to pain assessment?
3. How confident to you feel **talking with families** about assessing a patient’s pain or discomfort?
4. How confident do you feel **providing family members with information or education** about pain assessment?
5. How confident do you feel **providing family members with concrete strategies or steps** they can implement to assess their loved one’s pain?

Pain Management

1. How confident do you feel **managing** a patient’s pain?
2. How confident do you feel **assessing family member education needs** with respect to pain management?
3. How confident to you feel **talking with families** about managing a patient’s pain or discomfort in general?
4. How confident to you feel **talking with families** when medication problems arise?
5. How confident do you feel **talking with families** about using opioids such as morphine?
6. How confident do you feel **providing family members with information or education** about non-medication pain management?
7. How confident do you feel **providing family members with information or education** about medication to manage a patient’s pain?
8. How confident do you feel **providing family members with information or education** about morphine?
9. How confident do you feel **providing family members with concrete strategies or steps** that do not involve medication and they can implement to manage their loved one’s pain?
10. How confident do you feel **providing family members with concrete strategies or steps** involving medication and they can implement to manage their loved one’s pain?

Tool Video

In thinking about your home hospice patients living with dementia and their family members:

1. How confident do you feel **identifying family caregiver burden, stressors or worries** during routine home visits?
2. How confident do you feel **helping family caregivers** to address their burden or worries?
3. How confident do you feel **encouraging family caregivers to identify strategies** they can use to address their burden or worries?
4. How confident do you feel **identifying the pros and cons** of each strategy?
5. How confident do you feel **following up with family caregivers** about burden or worries?

(Post Tool video only)

1. After watching this video, how **confident** do you feel using the [assessment tool/worksheet] to assess family caregiver burden at initial and follow up visits?
2. How often do you plan to use the [tool/worksheet] to assess family caregiver burden?
   - - 1. Every visit
       2. Most visits
       3. Initial visit only
       4. Rarely
       5. Never (please explain)

Confidence Questions are asked all at once pre-video and immediately following each relevant video.

(responses: 1= not confident at all, 2, 3, 4, 5=somewhat confident, 6, 7, 8, 9, 10=very confident and 11 I do not conduct the hospice admission visit).

EHR Video

1. How confident do you feel recording family caregiver burden in the electronic hospice record at **admission visit**? [include option: I do not conduct the hospice admission visit].
2. How confident do you feel recording family caregiver burden in the electronic hospice record at **routine follow-up visits**?

(Pre video only)

1. How often do you record family caregiver burden at the admission visit?
2. Every visit
3. Most visits
4. Rarely
5. Never (please explain)
6. I do not conduct the hospice admission visit
7. How often do you record family caregiver burden during routine follow-up visits?
   1. Every visit
   2. Most visits
   3. Rarely
   4. Never (please explain)

(Post EHR video only)

1. How often do you plan to record family burden in the electronic hospice record?
   1. Every visit
   2. Most visits
   3. Initial visit only
   4. Rarely
   5. Never (please explain)

# Aim 2 and 3 Feasibility and Acceptability: User-Centered Design

**Family Care partners and Clinicians**

***Review of Individual Videos (Clinicians Only-to be administered post video training)***

Instructions: Now we are going to ask you some questions about each video. Thinking about the video on (*state topic*) (repeat for each video)

1. Did you have any issues accessing or viewing the video on [topic]?
   1. No
   2. Yes (please explain)
2. How much of this video did you watch?
   1. 25%
   2. 50%
   3. 75%
   4. All of it
   5. None of it
3. If 2b or 2c: I did not complete the videos because (check all that apply).

□ I had trouble understanding the videos

□ The videos were too long

□ I did not think the content of the videos was relevant to my work

□ I did not learn anything from the videos

□ I did not have time to watch the videos

□ Other (specify):

1. How helpful was the video on [topic] to you?
   1. Not at all helpful
   2. Slightly helpful
   3. Moderately helpful
   4. Helpful
   5. Very Helpful
2. Please indicate what you found helpful about the video:
3. How could we improve the video?
4. How difficult was it for you to understand the content of the video on [insert /topic] (e.g. language, ideas)?
   1. Very difficult
   2. Difficult
   3. Moderately difficult
   4. Slightly difficult
   5. Not at all difficult
5. [If participant scores >1 on the previous item]: I am going to list a few things that may have made the video difficult to understand. Indicate whether each option made the video difficult for you. Was the video difficult because the:
   1. Wording was too complicated: Y/N
   2. Ideas were too complicated: Y/N
   3. There was too much information: Y/N
   4. There was not enough information: Y/N
   5. Other (please specify): __________________________: Y/N
6. If you found the content too easy or too simple, what made it too easy?
7. What information was provided that will help you [support family members with respect to [patients' eating and drinking/pain/]/record family members’ burden in the electronic health record/assess family members' burden, stressors or worries]?
8. What additional information could be provided to help you better [support family members with respect to [patient’s' eating and drinking/pain/]/record family members’ burden in the electronic health record/support family members to reduce their burden, stressors or worries]?
9. What questions do you still have that would help you [support family members with respect to [patients' eating and drinking/pain/]/record family members’ burden in the electronic health record/support family members to reduce their burden, stressors or worries]?
10. How likely are you to use the information from the video on [topic] in your clinical practice?
    1. Very Unlikely
    2. Unlikely
    3. Neutral
    4. Likely
    5. Very likely

13a. Why or why not?

1. Overall, how satisfied are you with the video on [topic]?
   1. Very dissatisfied
   2. Dissatisfied
   3. Neutral
   4. Satisfied
   5. Very Satisfied

Instructions: Thinking about all of the videos, please answer the following questions.

*[questions 15-18 for aim 3 only]*

1. How did you watch the videos? Check all that apply.
   1. Phone
   2. Computer
   3. Tablet
   4. Other (please specify)
2. What is your preferred method of watching the videos?
   1. Phone
   2. Computer
   3. Tablet
   4. Other (please specify)
3. How difficult was it to view the videos using your preferred method?
   1. Very difficult
   2. Difficult
   3. Moderately difficult
   4. Slightly difficult
   5. Not at all difficult
4. Where were you when you were watching the videos? Check all that apply.
   1. At home
   2. Place of work
   3. During commute
   4. Car
   5. Other (please specify)
5. Who do you think it the appropriate audience for these videos? (check all that apply)
   1. Family members
   2. Hospice nurses
   3. Hospice social workers
   4. Other (please specify)
6. [If 15a]: When to you think the videos on eating and drinking and pain assessment are appropriate to share with family members?
7. Hospice admission visit
8. As issues related to eating and drinking and/or pain assessment arise
9. Other (please specify)
10. [If 15b or 15c] When do you think these videos are appropriate for hospice nurses and social workers to view? (check all that apply)
11. Onboarding for new hires
12. Annually
13. Twice a year
14. >2 times a year (specify how often)
15. These videos are not appropriate for hospice nurses and social workers
16. Thinking about the videos: there are 4 educational videos. Do you think that is:
    1. Too many videos
    2. An acceptable number of videos
    3. Not enough videos
17. If you could choose, what would be your preferred way to view the videos?
    1. On your own
    2. In a small group setting
    3. As part of clinical team meetings
    4. Other (please explain)
18. Which methods for viewing the videos would you be willing to do? (select all that apply)
    1. On your own
    2. In a small group setting
    3. As part of clinical team meetings
    4. Other (please explain)

***Review of [Assessment Tool/Worksheet] (Clinicians and FCP)***

Instructions: Please answer the following questions about the [assessment tool/worksheet] and ways we can make it better.

1. Did you have any issues accessing or viewing the [tool/worksheet]?
   1. No
   2. Yes (please explain)
2. How helpful was the [assessment tool/worksheet]?
   1. Not at all helpful
   2. Slightly helpful
   3. Moderately helpful
   4. Helpful
   5. Very Helpful
3. Please indicate what you found helpful about the [tool/worksheet].
4. How can we improve the [tool/worksheet]?
5. How difficult was it for you to understand the content of the [assessment tool/worksheet] (e.g. language, ideas)?
   1. Very difficult
   2. Difficult
   3. Moderately difficult
   4. Slightly difficult
   5. Not at all difficult
6. I am going to list a few things that may have made the video difficult to understand. Indicate whether each option made the video difficult for you. Was the video difficult because the:
   1. Wording was too complicated: Y/N
   2. Ideas were too complicated: Y/N
   3. There was too much information: Y/N
   4. There was not enough information: Y/N
   5. Other: __________________________: Y/N
7. If you found the content too easy or too simple, what made it too easy?
8. How helpful was the video in explaining how to use the [tool/worksheet]?
   1. Very helpful
   2. Somewhat helpful
   3. Neither helpful nor unhelpful
   4. Somewhat unhelpful
   5. Not at all helpful
9. Do you think it is necessary to watch the video in order to use the [tool/worksheet] correctly? Why or why not?
10. How confident would you be using the [tool/worksheet] without having seen the video?

a. Not confident at all

b. Not very confident

c. Somewhat confident

d. Confident

e. Very confident

Why do you think that?

1. [clinicians only] How likely are you to use the information [assessment tool/worksheet] in your clinical practice?
   1. Very Unlikely
   2. Unlikely
   3. Neutral
   4. Likely
   5. Very likely

11a. Why or why not?

1. Overall, how satisfied are you with the [assessment tool/worksheet]?
   1. Very dissatisfied
   2. Dissatisfied
   3. Neutral
   4. Satisfied
   5. Very Satisfied

***Review of Overall Intervention (Clinicians only)***

1. Overall, how helpful do you think the videos and [tool/worksheet] would be for you to help in providing care and support to your loved one or patients with memory problems?
   1. Not at all helpful
   2. Slightly helpful
   3. Moderately helpful
   4. Helpful
   5. Very Helpful
2. What are additional topics you think should be covered in the videos and [tool/worksheet]?
3. Regarding the amount of information in the videos ad [tool/worksheet], do you think there is:
   1. Too much information
   2. The right amount of information
   3. Not enough information
4. What additional information do you think should be included in the videos and [tool/worksheet]?
5. Overall, how satisfied are you with the intervention (videos and [assessment tool/worksheet] together)?
   1. Very dissatisfied
   2. Dissatisfied
   3. Neutral
   4. Satisfied
   5. Very Satisfied
6. Please provide any other general comments about the [videos and] [tool/worksheet].

# Aims 2 and 3. Feasibility and Acceptability.

***Post-intervention (to be completed at end of study enrollment)***

***Family Care partner***

1. How often did you use the [assessment tool/worksheet] with a hospice nurse or social worker?

4 times 3 times 2 times 1 time

□ I did not use the [assessment tool/worksheet] with a hospice nurse or social worker.

1. Which hospice team member used the [assessment tool/worksheet] with you?

□ Nurse

□ Social worker

□ Nurse and social worker

□ Don’t know

□ The hospice team did not use the [assessment tool/worksheet] with me

□ Other (specify):

1. About how long did it take you to use the [assessment tool/worksheet] with the hospice team member for the first time?

minutes

1. About how long did it take you to use the [assessment tool/worksheet] with the hospice team member during follow-up visits?

minutes

1. How helpful was the [assessment tool/worksheet]?
   1. Not at all helpful
   2. Slightly helpful
   3. Moderately helpful
   4. Helpful
   5. Very Helpful

5a. What about the [assessment tool/worksheet] did you find helpful?

5b. What about the [assessment tool/worksheet] did you find least helpful?

1. How difficult was it to use the [assessment tool/worksheet]?
   1. Very difficult
   2. Difficult
   3. Moderately difficult
   4. Slightly difficult
   5. Not at all difficult

6a. What about the [assessment tool/worksheet] was easy to use?

6b. What about the [assessment tool/worksheet] made it difficult to use?

1. Overall, how satisfied are you with the [assessment tool/worksheet]?
   1. Very dissatisfied
   2. Dissatisfied
   3. Neutral
   4. Satisfied
   5. Very Satisfied
2. What about the [assessment tool/worksheet] could we change to make you more likely to use it?

***Clinicians***

1. How often have you used any of the information in the educational videos in your work with home hospice patients with dementia and their families?

□ Frequently: with most of my patients with dementia

□ Sometimes: with some of my patients with dementia

□ Rarely: with a few of my patients with dementia

□ Never: I have not applied any of the information from the videos in my clinical practice

1. I used the information in the educational videos with home hospice patients without dementia:]

□ Yes

□ No

1. Which parts of the educational videos did you use in your clinical practice (check all that apply)?

□ Main point of video 1

□ Main point of video 2

□ Main point of video 3

□ Main point of video 4

□ Other (specify):

1. Overall, how satisfied are you with the educational videos?
   1. Very dissatisfied
   2. Dissatisfied
   3. Neutral
   4. Satisfied
   5. Very Satisfied
2. With how many FCP did you use the [assessment tool/worksheet]?

0 1 2 3 4 >4

□ I don’t have any patients with dementia on my caseload.

1. About how long did it take you to use the [assessment tool/worksheet] with a family member for the first time?

minutes

1. About how long did it take you to use the [assessment tool/worksheet] with a family member during follow-up visits?

minutes

1. How helpful was the [assessment tool/worksheet] when working with family members?
   1. Not at all helpful
   2. Slightly helpful
   3. Moderately helpful
   4. Helpful
   5. Very Helpful

7a. What about the [assessment tool/worksheet] did you find helpful when using with family members?

7b. What about the [assessment tool/worksheet] did you find least helpful when using with family members?

1. How difficult was it to use the [assessment tool/worksheet] as part of your clinical practice? (reverse code)
   1. Very difficult
   2. Difficult
   3. Moderately difficult
   4. Slightly difficult
   5. Not at all difficult

9a. What about the [assessment tool/worksheet] was easy to use?

9b. What about the [assessment tool/worksheet] made it difficult to use?

1. Overall, how many times did you use the worksheet with each family member?
   1. 4 times
   2. 1- 3 times
   3. Not at all
2. You were asked to use the worksheet up to four times with each family member enrolled in the study. Did you find that to be
   1. Not enough times
   2. The right amount of times
   3. Too many times

11a. [If 11 is A or C] What is the ideal number of times the worksheet should be used with each family member?

11b. [If 11 is A or C] Please explain?

1. How likely are you to continue to use the [assessment tool/worksheet], now that the study has concluded?
   1. Very Unlikely
   2. Unlikely
   3. Neutral
   4. Likely
   5. Very likely

12a. Why or why not?

1. What about the [assessment tool/worksheet] could we change to make you more likely to use it?
2. What were the types of barriers you observed to implementing the steps family members identified through the [assessment tool/worksheet]? (check all that apply)

□ Family members did not take steps they agreed upon (please explain)

□ Interdisciplinary team dynamics impacted family members from implementing steps (please explain)

□ Other (please explain)

1. Overall, how satisfied are you with the [assessment tool/worksheet]?
   1. Very dissatisfied
   2. Dissatisfied
   3. Neutral
   4. Satisfied
   5. Very Satisfied
2. Was there anything that happened during the study that took you by surprise?
   1. No
   2. Yes (please explain).
3. Overall, how satisfied are you with the intervention (videos and [assessment tool/worksheet] together)?
   1. Very dissatisfied
   2. Dissatisfied
   3. Neutral
   4. Satisfied
   5. Very Satisfied

**Evaluation Items for Continuing Education**

License number [Collected for individuals qualifying for continuing education for which license numbers are required]

Asked of all continuing education participants

*(repeated for each module)*

- 1. Indicate how you received this training:
     - 1. Live (in person or via zoom)
       2. Pre-recorded
       3. Online activity
     1. Please rate the speaker’s:
        1. Knowledge of subject
           - Excellent
           - Good
           - Fair
           - Poor
        2. Clarity of Content
           - Excellent
           - Good
           - Fair
           - Poor
        3. Effectiveness teaching
           - Excellent
           - Good
           - Fair
           - Poor
     2. Rate the degree to which the activity achieved stated learning objectives:
        1. Excellent
        2. Good
        3. Fair
        4. Poor

3a. Please explain [text box]

- - 1. Rate the content of the activity
       1. Excellent
       2. Good
       3. Fair
       4. Poor

4a. Please explain [text box]

- - 1. Rate the convenience of the schedule during which the activity was provided
       1. Excellent
       2. Good
       3. Fair
       4. Poor

Please explain [text box]

- - 1. Did you identify bias during the activity?
       1. No
       2. Yes (please explain)
    2. Overall how would you rate this activity?
       1. Excellent
       2. Good
       3. Fair
       4. Poor

## **FCP Post-Visit Fidelity Assessment Form** To be distributed to FCP following a visit with an enrolled clinician)

Our records indicate [insert enrolled clinician name] from VNS Health visited with your loved one on [insert date]. Thank you for answering the following question[s] about that visit.

1. [Control arm only] Please choose the option that best describes your interaction with [insert enrolled clinician name]:
   1. I did not speak with the clinician during the visit.
   2. We discussed what was happening with my loved one.
   3. We discussed what was bothering me.
   4. We discussed what was happening with my loved one and what was bothering me.
   5. Other (please explain)
2. [Intervention arm only]. Please choose the option that best describes your interaction with [insert enrolled clinician name].
   - - 1. I did not speak with [him/her/the nurse/the social worker/the clinician] during the visit.
       2. I spoke with [him/her/the nurse/the social worker/the clinician], but we did not discuss what was bothering me.
       3. We discussed what was bothering me and completed a worksheet together.
       4. We discussed what was bothering me and [she/he] left a worksheet for me to complete.
       5. We discussed what was bothering me.
       6. Other (please explain)

[Intervention arm only, if response to 2 is c or d] NOTE: final language will be adjusted to reflect words that are used to describe different components of the [tool/worksheet] based on feedback from Round 2 of Aim 1.

1. Did you have any issues completing the worksheet?
2. No
3. Yes (please explain)
4. Did you talk about different strategies to deal with what is bothering you?
   1. No
   2. Yes
5. Did you talk about pros and cons of the different strategies?
   1. No
   2. Yes
6. Do you decide on a strategy to follow?
   1. No
   2. Yes
7. Do you plan to follow the strategy?
   1. No
   2. Yes

Why or why not?

## **Clinician Post-Visit Fidelity Assessment Form** (To be distributed to clinicians following a visit with an enrolled FCP)

As part of your participation in the “Enhancing Dementia Instruction in Home Hospice Care (EDITH-HC)” study, please answer the following questions.

1. Please explain the main purpose of your visit on [insert date] with [insert patient name]?
2. [Control arm only] Please choose the option that best describes your interaction with [insert enrolled FCP name].
   1. We discussed what was happening with the patient.
   2. We discussed what was bothering the family member.
   3. We discussed what was happening with the patient and what was bothering the family member.
   4. I did not speak with the family member.
   5. Other (please explain)
3. [Intervention arm only] Please choose the option that best describes your interaction with [insert enrolled FCP name].
   1. The family member completed the [assessment tool/worksheet]] with some guidance from me.
   2. The family member completed the [assessment tool/worksheet]] with a lot of prompting from me.
   3. We completed the [assessment tool/worksheet]] together, making equal contributions.
   4. I left the [assessment tool/worksheet]] with the family member to complete on their own.
   5. I used the [assessment tool/worksheet]] as a guide, but did not complete it with the family member.
   6. We discussed what was bothering the family member, but I did not use the [assessment tool/worksheet]].
   7. I met with the family member, but we did not discuss what was bothering him/her.
   8. I did not speak with the family member during the visit.
   9. Other (please explain)
4. [Intervention arm only, if 3 d-f] Why didn’t you use the [assessment tool/worksheet] with the family member?
   1. I did not know I was supposed to do this.
   2. I forgot.
   3. I didn’t have a copy with me.
   4. There was not time to use it during the visit.
   5. The family member was not open to using the [tool/worksheet].
   6. This wasn’t an appropriate visit for the [tool/worksheet] to be used
   7. Other (please explain)
5. [intervention arm only if 3 a-c] Did you have any issues using the [assessment tool/worksheet] with the family member?
   1. No
   2. Yes (please explain)
6. [Intervention arm only, if 3 a-c] What challenge or concern did the family member identify? [text box]
7. [Intervention arm only] Did you record family member burden in the electronic hospice record following your visit?
   1. Yes
   2. No
8. [Intervention arm only if 6 b] Why didn’t you record family member burden in the electronic hospice record?
   1. I did not know I was supposed to do this.
   2. I do now know how to do this.
   3. I forgot.
   4. I did not have time.
   5. This wasn’t an appropriate visit for me to ask questions related to burden
   6. Other (please explain)
9. [Intervention arm only if 6 a]. Did you have any issues recording family member burden in the electronic hospice record?
   1. No
   2. Yes (please explain)
10. Additional comments or explanation.

| ***Table 2. Additional Feasibility and Fidelity Measures*** |  |
| --- | --- |
| **Feasibility and Fidelity Items** | **Measurements** |
| 1. Accrual rates | Number of potential participants contacted,  Percent of contacted enrolled  Number enrolled |
| 2. Attrition rates | Number enrolled,  Number and percent dropped out |
| 3. Adherence to the study protocol | Number and percent of clinicians:  1. viewing3 or more of the videos,  2. viewing more than 80% of each video,  3. using the tool more than 75% of the time,  4. number and percent of family care partners using the tool,  5. number of times family care partners are using the tool |
| 4. Number of educational video views | Total number of views,  Total number of unique viewers |
| 5. Frequency of issues related to viewing videos | Number of times clinicians report issues watching the video |
| 6. Types of issues related to viewing videos | Issues described by clinicians |
| 7. Number of assessment tool initial uses | Number of care partners with whom clinicians use the tool |
| 8. Number of assessment tool follow-up uses | Number of times clinicians use the tool as a follow-up |
| 9. Frequency of issues using assessment tool | Number of times clinicians report issues using the tool,  Number of times family care partners report issues using the tool |
| 10. Types of issues using assessment tool | Types of issues described by clinicians,  Types of issues described by family care partners |
| 11. Number of EHR burden reports | Number of times family care partner burden is recorded in the EHR from enrolled care partners |
| 12. Frequency of issues using EHR burden reports | Number of times clinicians report burden in the EHR |
| 13. Types of issues using EHR burden reports | Types of issues clinicians report recording burden in the EHR |
| 14. Number of clinicians using assessment tool | Number and percent of enrolled clinicians using the assessment tool |
| 15. Number of FCP with whom assessment tool is used | Number and percent of enrolled family care partners using the assessment tool as reported by family care partners and clinicians |
| 16. Number of data collection points with FCP | Total number of data collection points with family care partners  Average number of data collection points with family care partners (0-4),  Percent of family care partners with complete data for their hospice experience |
| 17. Frequency of issues completing data collection with FCP | Number of times issues with data collection are recorded for family care partners |
| 18. Types of issues with completing data collection with FCP | Types of issues with data collection recorded for family care partners |
| 19. Number of successful post-intervention surveys with clinicians | Number and percent of enrolled clinicians completing post intervention survey |
| 20. Frequency of issues completing post-intervention surveys with clinicians | Number of times with data collection that are recorded for clinicians |
| 21. Types of issues with completing post-intervention surveys with clinicians | Types of issues with data collection recorded for clinicians |

# Aim 3. Clinician assessment tools (Pre/Post, intervention and control)

***Dementia knowledge assessment scale***

Annear, M. J., Toye, C. M., Eccleston, C. E., McInerney, F. J., Elliott, K. E. J., Tranter, B. K., ... & Robinson, A. L. (2015). Dementia knowledge assessment scale: development and preliminary psychometric properties. *Journal of the American Geriatrics Society*, *63*(11), 2375-2381.

| **Q #** | **Statements about dementia** | **Response scale**  (Please tick one box ) | | | |  |
| --- | --- | --- | --- | --- | --- | --- |
|  |  | **False** | **Probably false** | **Probably true** | **True** | **I don’t know** |
| A1 | Dementia is a normal part of the ageing process. |  |  |  |  |  |
| A2 | Alzheimer’s disease is the most common form of dementia. |  |  |  |  |  |
| A3 | People can recover from the most common forms of dementia. |  |  |  |  |  |
| A4 | Dementia does **not** result from physical changes in the brain. |  |  |  |  |  |
| A5 | Planning for end of life care is generally **not** necessary  following a diagnosis of dementia. |  |  |  |  |  |
| A6 | Blood vessel disease (vascular dementia) is the most common form of dementia. |  |  |  |  |  |
| A7 | Most forms of dementia do **not** generally shorten a person’s life. |  |  |  |  |  |
| A8 | Having high blood pressure increases a person’s risk of developing dementia. |  |  |  |  |  |
| A9 | Maintaining a healthy lifestyle does **not** reduce the risk of  developing the most common forms of dementia. |  |  |  |  |  |
| A10 | Symptoms of depression can be mistaken for symptoms of dementia. |  |  |  |  |  |
| A11 | Exercise is generally beneficial for people experiencing  dementia. |  |  |  |  |  |
| A12 | Early diagnosis of dementia does **not** generally improve quality of life for people experiencing the condition. |  |  |  |  |  |
| A13 | The sudden onset of cognitive problems is characteristic of  common forms of dementia. |  |  |  |  |  |
| A14 | It is impossible to communicate with a person who has advanced dementia. |  |  |  |  |  |
| A15 | A person experiencing advanced dementia will **not** generally  respond to changes in their physical environment. |  |  |  |  |  |
| A16 | It is important to correct a person with dementia when they are confused. |  |  |  |  |  |
| A17 | People experiencing advanced dementia often communicate through body language. |  |  |  |  |  |
| A18 | Uncharacteristic behaviours in a person experiencing dementia are generally a response to unmet needs. |  |  |  |  |  |
| A19 | Medications are the most effective way of treating behavioural symptoms of dementia. |  |  |  |  |  |
| A20 | People experiencing dementia do **not** generally have problems making decisions. |  |  |  |  |  |
| A21 | Movement is generally affected in the later stages of dementia. |  |  |  |  |  |
| A22 | People with advanced dementia may have difficulty  speaking. |  |  |  |  |  |
| A23 | People experiencing dementia often have difficulty learning new skills. |  |  |  |  |  |
| A24 | Difficulty eating and drinking generally occurs in the later stages of dementia. |  |  |  |  |  |
| A25 | Daily care for a person with advanced dementia is effective when it focuses on providing comfort. |  |  |  |  |  |

**DKAS total scoring**

Step one: In your database, DKAS response categories should be labelled as follows:

False 1

Probably false 2

Probably true 3

True 4

I don’t know 5

Step two: Enter DKAS data.

Step three: Recode responses to ‘false’ statements and apply scoring system. The DKAS scoring system is as follows:

 Score 2 points for an answer of ‘true’ to a truthful statement.

 Score 2 points for an answer of ‘false’ to an untrue statement.

 Score 1 point for an answer of ‘probably true’ to a truthful statement.

 Score 1 point for an answer of ‘probably false’ to an untrue statement.

 Score 0 points for an answer of ‘true’ or ‘probably true’ to an untrue statement.

 Score 0 points for an answer of ‘false’ or ‘probably false’ to a truthful statement.

 Score 0 points for an answer of ‘I don’t know’.

Step four: Sum items to provide a total score. The maximum total score on the DKAS v2.0 is **50**.

Step five: To calculate subscale scores, sum the items for each subscale (see Annear et al. 2017 for subscale items). In order to compare the subscales, you may wish to standardize each subscale to a maximum of 1.

# Aim 3. FCP assessment tools (baseline, follow-up visits 2-4, intervention and control)

***Terminal Illness Acceptance***

1. Describe your loved one’s current health status

□ Relatively healthy

□ Seriously but not terminally ill

□ Seriously and terminally ill

***General Preparedness***

2. To what extent do you feel prepared for your loved one’s death? (Herbert, et al 2006 REACH study)

□ Not at all

□ Somewhat

□ Very much

***Self-Administered Caregiving Self-Efficacy Scale (CSES-8)***

We are interested in how sure you are that you can keep up your own activities and also respond to caregiving situations for your care partner (the person you care for). For each of the following questions, please ***circle*** the number that corresponds **how sure** you are that you can do the tasks regularly **at the present time**.

| 1. How sure or confident are you that you can ask a friend/family member to stay with your care partner for a day when you need to see the doctor yourself? | ________________________________  Not at all \| \| \| \| \| \| \| \| \| \| Totally  confident 1 2 3 4 5 6 7 8 9 10 confident |
| --- | --- |
| 2. How sure or confident are you that you can stop yourself from thinking about unpleasant aspects of taking care of your care partner? | ________________________________  Not at all \| \| \| \| \| \| \| \| \| \| Totally  confident 1 2 3 4 5 6 7 8 9 10 confident |
| 3. How sure or confident are you that you can stop yourself from worrying about future problems that might come up with your care partner? | ________________________________  Not at all \| \| \| \| \| \| \| \| \| \| Totally  confident 1 2 3 4 5 6 7 8 9 10 confident |
| 4. How sure or confident are you that you can cope with unexpected or new situations that may come up with your care partner? | ________________________________  Not at all \| \| \| \| \| \| \| \| \| \| Totally  confident 1 2 3 4 5 6 7 8 9 10 confident |
| 5. How sure or confident are you that you can do the things necessary to keep your stress under control? | ________________________________  Not at all \| \| \| \| \| \| \| \| \| \| Totally  confident 1 2 3 4 5 6 7 8 9 10 confident |
| 6. How sure or confident are you that you can do the things necessary to take care of your own health? | ________________________________  Not at all \| \| \| \| \| \| \| \| \| \| Totally  confident 1 2 3 4 5 6 7 8 9 10 confident |
| 7. How sure or confident are you that you can find resources in the community (meals, legal, support groups, etc.) that can help you take care of yourself and your care partner? | ________________________________  Not at all \| \| \| \| \| \| \| \| \| \| Totally  confident 1 2 3 4 5 6 7 8 9 10 confident |
| 8. Sometimes prevent your care partner from becoming angry or disruptive? | ________________________________  Not at all \| \| \| \| \| \| \| \| \| \| Totally  confident 1 2 3 4 5 6 7 8 9 10 confident |

***Preparedness Scale of the Family Care Inventory***

We know that people may feel well prepared for some aspects of giving care to another person, and not as well prepared for other aspects. We would like to know how well prepared you think you are to do each of the following, even if you are not doing that type of care now.

|  | Not at all prepared | Not too well prepared | Somewhat well prepared | Pretty well prepared | Very well prepared |
| --- | --- | --- | --- | --- | --- |
| 1. How well prepared do you think you are to find out about and set up services for him or her? | 0 | 1 | 2 | 3 | 4 |
| 1. How well prepared do you think you are for the stress of caregiving? | 0 | 1 | 2 | 3 | 4 |
| 1. How well prepared do you think you are to take care of his or her emotional needs? | 0 | 1 | 2 | 3 | 4 |
| 1. How well prepared do you think you are to find out about and set up services for him or her? | 0 | 1 | 2 | 3 | 4 |
| 1. How well prepared do you think you are for the stress of caregiving? | 0 | 1 | 2 | 3 | 4 |
| 1. How well prepared do you think you are to make caregiving activities pleasant for both you and your family member? | 0 | 1 | 2 | 3 | 4 |
| 1. How well prepared do you think you are to respond to and handle emergencies that involve him or her? | 0 | 1 | 2 | 3 | 4 |
| 1. How well prepared do you think you are to get the help and information you need from the health care system? | 0 | 1 | 2 | 3 | 4 |
| 1. Overall, how well prepared do you think you are to care for your family member? | 0 | 1 | 2 | 3 | 4 |

Is there anything specific you would like to be better prepared for?__________________________________________________________________________________ __________________________________________________________________________________________________________________________________________________________________________ _____________________________________________________________________________________

MEAN SCORE of the number of items answered: ___________

***Zarit Burden Interview-12 item***

| Do you feel/wish… | Never | Rarely | Sometimes | Quite frequently | Nearly always |
| --- | --- | --- | --- | --- | --- |
| 1. You don’t have enough time for yourself? | 0 | 1 | 2 | 3 | 4 |
| 1. Stressed between caring and meeting other responsibilities? | 0 | 1 | 2 | 3 | 4 |
| 1. Angry when around your relative? | 0 | 1 | 2 | 3 | 4 |
| 1. Your relative affects your relationship with others in a negative way? | 0 | 1 | 2 | 3 | 4 |
| 1. Strained when you are around your relative? | 0 | 1 | 2 | 3 | 4 |
| 1. Your health has suffered because of your involvement with your relative? | 0 | 1 | 2 | 3 | 4 |
| 1. You don’t have as much privacy as you would like, because of your relative? | 0 | 1 | 2 | 3 | 4 |
| 1. Your social life has suffered because you are caring for your relative? | 0 | 1 | 2 | 3 | 4 |
| 1. You have lost control of your life since your relative’s illness?? | 0 | 1 | 2 | 3 | 4 |
| 1. Understanding about what to do about your relative | 0 | 1 | 2 | 3 | 4 |
| 1. You should be doing more for your relative? | 0 | 1 | 2 | 3 | 4 |
| 1. You could do a better job in caring for your relative? | 0 | 1 | 2 | 3 | 4 |

# Aim 3. Electronic Health Record Data (post intervention, intervention and control)

1. Patient age, gender, race/ethnicity, and whether dementia is a primary or comorbid diagnosis
2. Dates of hospice enrollment and discharge
3. Hospice discharge status
4. Dates of hospice clinician visits
5. Clinician visits (e.g. physician, nurse, social worker; study participants will be identified by study identifier)
6. Duration of hospice clinician visits
7. Dates of care partner burden assessments
8. Clinician making burden assessment (e.g. physician, nurse, social worker; study participants will be identified by study identifier)
9. Care partner burden assessment scores
